# Pharmacogenetics as a Future Tool to Risk-Stratify Breast Cancer Patients According to Chemotoxicity Potential from the Doxorubicin Hydrochloride and Cyclophosphamide (AC) Regimen

**DOI:** 10.3390/ph18040539

**Published:** 2025-04-07

**Authors:** Esraa K. Abdelfattah, Sanaa M. Hosny, Amira B. Kassem, Hebatallah Ahmed Mohamed Moustafa, Amany M. Tawfeik, Marwa N. Abdelhafez, Wael El-Sheshtawy, Bshra A. Alsfouk, Asmaa Saleh, Hoda A. Salem

**Affiliations:** 1 Department of Clinical Pharmacy, Faculty of Pharmacy (Girls), Al-Azhar University, Cairo 11651, Egypt; esraa.12147el@azhar.edu.eg (E.K.A.); sanaamohsen.2020@azhar.edu.eg (S.M.H.); 2 Clinical Pharmacy and Pharmacy Practice Department, Faculty of Pharmacy, Damanhour University, Damanhour 22514, Egypt; 3 Clinical Pharmacy and Pharmacy Practice Department, Faculty of Pharmacy, Badr University in Cairo, Cairo 11829, Egypt; 4 Medical Microbiology and Immunology Department, Faculty of Medicine, Badr University in Cairo, Cairo 11829, Egypt; amany-mohamed1@buc.edu.eg; 5 Medical Microbiology and Immunology Department, Faculty of Medicine, Al Azhar University (Girls) Cairo, Cairo 11754, Egypt; 6 Department of Medical Oncology, National Cancer Institute, Cairo University, Al Giza 12613, Egypt; marwa.nabill@nci.cu.edu.eg; 7 Department of Medical Oncology, Faculty of Medicine, Al Azhar University, Cairo 11651, Egypt; wael.el-sheshtawy@azhar.edu.eg; 8 Department of Pharmaceutical Sciences, College of Pharmacy, Princess Nourah bint Abdulrahman University, P.O. Box 84428, Riyadh 11671, Saudi Arabia; baalsfouk@pnu.edu.sa (B.A.A.); asali@pnu.edu.sa (A.S.); 9 Department of Pharmacy Practice, University of Tabuk, Tabuk 47512, Saudi Arabia; hsalem@ut.edu.sa

**Keywords:** single-nucleotide polymorphism, SNP, AC regimen, rs2228100, rs12248560, rs1045642, rs6907567

## Abstract

**Background:** Studying single-nucleotide polymorphisms (SNPs) in xenobiotic-transporting and metabolizing enzyme genes before administering the doxorubicin hydrochloride and cyclophosphamide (AC) regimen may help optimize breast cancer (BC) treatment for individual patients. **Objective**: Genotyping specific SNPs on genes encoding for the transport and metabolism of the AC regimen and study their association with its chemotherapeutic toxicity. **Method:** This prospective cohort study was conducted in two hospitals in Egypt. Before receiving AC therapy, venous blood was collected from female patients with BC for DNA extraction and the genotyping of four SNPs: rs2228100 in *ALDH3A1* gene, rs12248560 in *CYP2C19* gene, rs1045642 in *ABCB1* gene, and rs6907567 in *SLC22A16* gene. Patients were then prospectively monitored for hematological, gastrointestinal, and miscellaneous toxicities throughout the treatment cycles. **Results:** The *ALDH3A1* gene polymorphism demonstrated a significant increase in nausea, stomachache, and peripheral neuropathy among patients carrying the GC+CC genotype, compared to those with the GG genotype (*p* = 0.023, 0.036, and 0.008, respectively). Conversely, patients with the GG genotype exhibited significantly higher fever grades after cycles 1, 2, and 3 of the AC regimen compared to those with the GC+CC genotype (*p* = 0.009, 0.017, and 0.018, respectively). Additionally, fatigue severity was significantly increased among patients with the GG genotype compared to those with the GC+CC genotype following AC administration (*p* = 0.008). **Conclusions:** The SNP variation of *ALDH3A1* (rs2228100) gene significantly influenced AC regimen toxicity in female BC patients. Meanwhile, SNPs in *CYP2C19* (rs12248560), *ABCB1* (rs1045642), and *SLC22A16* (rs6907567) genes showed a significant influence on the recurrence rate of certain toxicities.

## 1. Introduction

Breast cancer (BC) is the most frequent cancer among females, with substantial morbidity and mortality, making it a primary public health concern [1,2]. The majority of breast cancers are adenocarcinomas, primarily categorized into ductal and lobular types [3]. In addition, BC onset is influenced by mutations in BRCA1 and BRCA2 genes [2,4]. Invasive forms of the disease are classified as infiltrative, whereas non-infiltrative cases are referred to as “in situ” carcinoma [5]. Risk factors for BC development include smoking, alcohol intake, obesity, advanced age, exposure to radiation, and use of hormone replacement therapy [2,6,7].

BC treatment involves a combination of local modalities, such as surgery and radiotherapy, along with pharmacological approaches, including hormonal therapy (e.g., anti-estrogens and aromatase inhibitors), chemotherapy (e.g., cyclophosphamide, anthracycline drugs, platinum-based drugs, and taxanes), and targeted therapy, with variable response in practice [8,9]. Therapeutic decisions on BC are based on its three main subtypes, luminal (A and B), which express the hormone receptor such as estrogen receptor or progesterone receptor); human epidermal growth factor receptor 2-positive; and triple-negative, which lacks the expressions of all three receptors (estrogen receptor, progesterone receptor, and human epidermal growth factor receptor 2) [10]. Additional factors, including age at diagnosis, tumor histotype, and tumor-node-metastasis (TNM) staging, also play a crucial role in determining the appropriate treatment strategy [11].

Despite advancements in BC therapies, drug resistance and treatment-related toxicities remain significant challenges that can limit treatment success and overall survival [12,13]. Several clinical factors, including molecular subtype [14,15,16], receptor status [17], age [18], and tumor size [19] correlate with the response to chemotherapy. However, even among patients with similar clinical and pathological characteristics, genetic variations may significantly influence individual treatment outcomes [13,20]. In addition, subjects may develop heterogenicity in toxicities after the administration of equal doses [12,20,21,22], further complicating treatment management.

Chemotherapy is integral to the standardized systemic management of BC. It can be used as a neoadjuvant to diminish tumor size before surgery or as an therapy in early-stage BC cases with a favorable prognosis [23,24,25]. The toxicities of the chemotherapy may affect the quality of life of the individual and necessitate dose reductions or cycle delays, which may ultimately compromise the efficacy of the therapy [26]. Some patients may even refuse subsequent chemotherapy cycles due to concerns about adverse effects, increasing the risk of cancer recurrence or premature death [27,28]. Therefore, it is essential for patients receiving chemotherapy to be fully informed about both its potential benefits and the associated acute and long-term toxic effects [29].

The AC regimen was associated with an elevated risk of multiple toxicities, including gastrointestinal toxicities, such as nausea and vomiting, as well as hematological toxicities, such as neutropenia and leukopenia [30,31]. Pharmacogenetics is a science that studies genetically determined variations in pharmacokinetics including the absorption, distribution, metabolism, and excretion (ADME) of a drug. While demographic, clinical, concurrent medication, and environmental factors influence drug efficacy and toxicity, pharmacogenetics studies specifically associate the efficacy and toxicity of drugs with genetic variations among subjects [32,33].

Pharmacogenetic polymorphism is a monogenic trait arising from the presence of multiple alleles at the same genetic locus, leading to phenotypic variability in drug response within a population. The frequency of the least common allele exceeds 1%, influencing the medication metabolism, efficacy, and safety. Variations in DNA sequences may present as nucleotide insertions or deletions, alterations in the copy number of repetitive sequences, or single-nucleotide polymorphisms (SNPs) [34,35]. Incorporating these SNPs into clinical procedures allows for informed decision-making, enabling clinicians to personalize chemotherapy regimens and optimize the treatment outcomes [36].

Pharmacokinetics or pharmacodynamics (drug actions on the body) play a critical role in determining the response to chemotherapeutic drugs. These processes rely on receptor metabolizing and transporter proteins encoded with various genes [8,37,38]. Genetic polymorphisms influence both the onset and progression of cancer, as well as the efficacy and adverse effects of chemotherapy [8,39]. Although pharmacogenetic research on chemotherapeutic toxicities is important for patients’ quality of life and survivorship, studies are still sparse [12,31,40,41]. While some drug–gene correlations have strong or moderate evidence, many remain inconclusive, with low levels of supporting data [8,42].

Alterations in the genes encoding drug metabolizing enzymes can influence their activities and expression, with subsequent differences in treatment outcomes [43]. The majority of published research examined the correlation between genetic polymorphisms and clinical outcomes of BC, including disease-free survival (DFS) and overall survival (OS) [44,45], with scarce evidence on treatment response and toxicity [25,46]. Expanding this research area could contribute to more personalized and effective cancer treatment strategies.

Phase I CYP450, *CYP2C19* is one of multiple metabolizing isoenzymes catalyzing the oxidation of the pro-drug, cyclophosphamide, into its active form [25,47,48], which diffuses into cancer cells and exerts cytotoxic effects through alkylation [49,50]. CYP2C19*17 genetic polymorphism alters enzymatic expression and metabolism between individuals [51,52] leading to the ultra-rapid metabolism of *CYP2C19* substrates [53]. CYP2C19*17 (-806 C>T, or rs12248560) is an SNP that is characterized by high enzyme activities due to increased gene transcription, arising from the attachment of a specific nuclear protein to the 5′ flanking region [52]. It was detected in about 18% of African populations and 18-28% of Europeans, and it was less common in Asians [52]. Due to its exceptional rapid clearance function, the CYP2C19*17 allele can justify the lack of response to common doses of some proton pump inhibitors and antidepressants [54].

Aldehyde dehydrogenase (ALDH) family genes metabolize the toxic cyclophosphamide [41,55]. *ALDH3A1* belongs to the aldehyde dehydrogenase 3 family and it is located on chromosome 17p11.2 [56]. It metabolizes endogenous and exogenous aldehydes involved in cell division, migration, and oxidative stress response [57]. The rs2228100 polymorphism is positioned at the 8th exon of *ALDH3A1* gene. It is a missense mutation (P329A), where C allele is the minor (rare) variant, the G allele is the major (common) variant (GG genotype representing the wild-type) [58].

SNPs can lead to genetic variations that influence the expression and activity of transporters and enzymes, impacting the AC regimen response and tolerance [25,28,59,60]. The transport systems, including importers and exporters, regulate intracellular drug concentrations and modulate the treatment response and toxicity development [31]. *SLC22A16* [61] and *ABCB1* genes, also referred to as MDR1; P-gp [62] code for the influx and efflux of doxorubicin, respectively, influencing its systemic pharmacology [25,63].

*SLC22A16* encodes a membrane uptake transporter that facilitates doxorubicin entry into cells [25]. The overexpression of *SLC22A16* in cancer cells enhances their sensitivity to the cytotoxic effects of doxorubicin [48,61]. rs6907567 is a missense SNP of *SLC22A16* [64] which is located at the second exon having A>G alleles; in the other words, A is the major [25,65] and G is the altered (mutated) allele. Patients carrying the common A allele may be at a higher risk for neutropenia and require dose delay when they receive cyclophosphamide, doxorubicin, and fluorouracil, compared to the AG and GG genotypes [25].

ATP-binding cassette, subfamily B, member 1 (ABCB1) is a major efflux p-glycoprotein transporter for doxorubicin. It belongs to the ATC-binding cassette family, which protects normal cells against environmental toxins, by the pumping-out mechanism, causing their excretion into the bile [25,63,66]. It transports doxorubicin via ATP hydrolysis across the membrane, opposing the concentration gradient and plays a role in the resistance to doxorubicin [63]. SNPs in *ABCB1* were related to the doxorubicin concentration [67], and its removal from BC cells, affecting its effectiveness [11,30]. The overexpression of efflux transporters enhances drug elimination, potentially mitigating drug-induced harm [68].

At present, there are no established techniques for identifying patients at increased risk of chemotherapy-related toxicities before AC therapy administration. Consequently, all patients receiving the AC regimen must be meticulously monitored by their physician [69]. Identifying polymorphisms in genes coding for AC regimen transporter and metabolizing enzymes before the initiation of the AC regimen may present novel opportunities for individualized treatment optimization.

This is, to our knowledge, the first study in Egypt that evaluates the effect of SNPs in metabolic enzymes genes *CYP2C19* (rs12248560) and *ALDH3A1* (rs2228100), which play a role in the metabolism of cyclophosphamide drug in an AC regimen in female BC patients, along with SNPs in two transporter genes *ABCB1* (rs1045642) and *SLC22A16* (rs6907567), on the occurrence of adverse effects. Understanding these genetic variations could be valuable in predicting individual susceptibility to AC regimen-related toxicities, paving the way for personalized BC treatment strategies.

## 2. Results

### 2.1. Count and Percentage of Each Genotype Group Among the 96 Patients Along the CYP2C19, ALDH3A1, SLC22A16, and ABCB1 Genes

Among the 106 Egyptian female patients diagnosed with breast cancer who met the inclusion criteria, only 96 cases provided written consent to participate. Of these, 64 patients carried the CC genotype (66.67%) of the *CYP2C19* metabolizing enzyme gene, 28 carried the CT genotype (29.17%), and only 4 carried the TT genotype (4.17%). When combining the CT and TT genotypes, 32 patients (33.33%) carried the CT+TT genotypes. For the *ALDH3A1* metabolizing enzyme gene, 46 patients carried the GG genotype (47.92%), 39 carried the GC genotype (40.63%), and 11 carried the CC genotype (11.46%). When combining the GC and CC genotypes, 46 patients (47.92%) had the GC+CC genotypes. Regarding the SLC22A16 gene, 47 patients (48.96%) carried the AA genotype, 38 carried the GA genotype (39.58%), and 11 carried the GG genotype (11.46%). When combining the GG and GA genotypes, 49 patients (51.04%) had the GG+GA genotypes. For the *ABCB1* gene, 47 patients (48.96%) carried the GG genotype, 11 carried the AA genotype (11.46%), and 38 carried the GA genotype (39.58%). When combining the GA and AA genotypes, 47 patients (48.96%) had the GA+AA genotypes (Table A1).

### 2.2. Demographic Data Analysis

#### 2.2.1. General Demographic Data

Quantitative general demographic variables included age, body surface area, number of positive lymph nodes, and the number of lymph nodes removed. No significant differences were observed between the allele groups for the *CYP2C19*, *ALDH3A1*, *SLC22A16*, and *ABCB1* genes (*p*-value > 0.05) (Table 1). Qualitative general demographic variables, including marital status, menopausal status, oral contraception, family history, and smoking history, also showed no significant differences across the allele groups of *CYP2C19, ALDH3A1*, and *SLC22A16* genes (*p*-value > 0.05). However, a significantly higher proportion of married patients was observed in the GG genotype group of the *ABCB1* gene compared to the AA+GA group (*p*-value < 0.05) (Table 1).

#### 2.2.2. Surgical Demographic Data

Qualitative surgical demographic variables, including the type of surgery, affected breast, tumor grade, tumor stage (T stage, N stage), and adjuvant/neoadjuvant treatment setting, showed no significant differences among the allele groups of *CYP2C19*, *ALDH3A1*, *SLC22A16*, and *ABCB1* genes (*p*-value > 0.05) (Table 1).

#### 2.2.3. Pathological Demographic Data

Qualitative pathological variables, including pathological breast cancer type, progesterone receptor status, estrogen receptor status, and human epidermal growth factor receptor 2 status, showed no significant correlation with the different allele groups of *CYP2C19*, *ALDH3A1*, *SLC22A16*, and *ABCB1* genes demonstrated no significant difference (*p*-value > 0.05) (Table 1).

### 2.3. Toxicity Analysis

#### 2.3.1. *CYP2C19*, *ALDH3A1*, *SLC22A16*, and *ABCB1* Genes Data Analysis Related to Toxicity Assessed at Different Time Points

##### *CYP2C19*, *ALDH3A1*, *SLC22A16*, and *ABCB1* Genes Data Analysis Related to Hematological Toxicity Assessed at Different Time Points

Qualitative hematological toxicity variables, including thrombocytopenia, leukopenia, neutropenia, lymphocytopenia, and anemia, were assessed at five different time points for their correlation with various alleles of the *CYP2C19*, *ALDH3A1*, *SLC22A16*, and *ABCB1* genes. No significant differences were observed among the allele groups (*p*-value > 0.05) (Table 2, Table 3, Table 4 and Table 5).

##### *CYP2C19*, *ALDH3A1*, *SLC22A16*, and *ABCB1* Genes Data Analysis Related to Gastrointestinal (GIT) Toxicity at Different Time Points

The correlation between qualitative gastrointestinal (GIT) toxicity variables (nausea, vomiting, diarrhea, constipation, mucositis, and stomachache) and different alleles of the *CYP2C19* gene at five time points showed no significant differences between allele groups (*p*-value > 0.05) (Table 6). However, as shown in Table 7, the correlation of these GIT toxicity variables with different alleles of the *ALDH3A1* gene revealed a significant increase in nausea among patients carrying the GC+CC genotype compared to those with the GG genotype after the third cycle of the AC regimen (*p* = 0.023). Additionally, a significant increase in stomachache was observed among patients with the GC+CC genotype versus the GG genotype after the third cycle of the AC regimen (*p* = 0.036).

Conversely, no significant differences were observed between the *ALDH3A1* gene and its different alleles concerning other GIT toxicity variables (*p*-value > 0.05). Similarly, there was no significant difference in GIT side effects between the patients carrying the GG+GA genotype and those with the AA genotype of the *SLC22A16* gene, as shown in Table 8.

There was no significant difference in gastrointestinal side effects between patients carrying the AA+GA genotype and those carrying the GG genotype of the *ABCB1* gene (Table 9).

##### The *CYP2C19*, *ALDH3A1*, *SLC22A16*, and *ABCB1* Genes Data Analysis Related to Miscellaneous Toxicity at Different Time Points

Qualitative miscellaneous toxicity variables, including fever, fatigue, amenorrhea, alopecia, headache, skin toxicity, and peripheral neuropathy, were assessed across five time points. The correlation between these variables and different alleles of the *CYP2C19* gene showed no significant differences between the allele groups (*p*-value > 0.05) (Table 10).

As presented in Table 11, the correlation between qualitative miscellaneous toxicities and different alleles of the *ALDH3A1* gene revealed a significant increase in fever among patients carrying the GG genotype compared to those with the GC+CC genotype after the first, second, and third cycles of the AC regimen (*p* = 0.009, *p* = 0.017, and *p* = 0.018, respectively). Additionally, there was a significant increase in fatigue among patients carrying the GG genotype, versus the GC+CC genotype after cycle one of the AC regimen (*p* = 0.008). Peripheral neuropathy also showed a significant increase in patients carrying the GC+CC genotype compared to those with the GG genotype after the second cycle of the AC regimen (*p* = 0.008). However, no significant differences were observed between *ALDH3A1* alleles concerning other toxicity variables (*p*-value > 0.05).

Analysis of qualitative data for miscellaneous toxicities showed no significant differences between the allele groups of the *SLC22A16* transporter gene (Table 12) or the *ABCB1* transporter gene (Table 13).

#### 2.3.2. Toxicity Among the Same Genotype Group Data Analysis Using Two-Way with One-Repeated-Measure ANOVA Test Results

##### Hematological Toxicity Among the Same Genotype Group Data Analysis Using Two-Way with One-Repeated-Measure ANOVA Test Results

A two-way analysis of variance (ANOVA) using the fit general linear model was conducted to assess the correlation between different genotypes and the quantitative hematological toxicity variables, including thrombocytopenia, leukopenia, neutropenia, lymphocytopenia, and anemia grades. The analysis revealed a significant increase in the mean leukopenia and lymphocytopenia grades after the fourth cycle of the AC regimen compared to baseline (point A) among patients carrying the CC genotype of the *CYP2C19* gene (*p* < 0.05). Additionally, the neutropenia grade significantly increased following the second and fourth cycles of chemotherapy in the same genotype group (*p*-value < 0.05).

Similarly, among patients with the GG genotype of the *ALDH3A1* gene, the mean anemia grade showed a significant increase after the fourth cycle of the AC regimen compared to baseline (*p* < 0.05). Furthermore, thrombocytopenia grades significantly increased after the first, second, and third cycles of chemotherapy, while neutropenia grades were significantly elevated after the first and third cycles in the same genotype group (*p* < 0.05). Patients carrying the GC+CC genotype of the *ALDH3A1* gene also demonstrated a significant increase in thrombocytopenia grades after the first, second, and third cycles of the AC regimen (*p*-value < 0.05).

Regarding the *SLC22A16* gene, the neutropenia grade increased significantly following the second and fourth cycles, while the lymphocytopenia grade increased significantly after the fourth cycle among patients with the AA genotype (*p* < 0.05). Among patients carrying the GG+GA genotype of the *SLC22A16* gene, leukopenia grade significantly increased after the fourth cycle, and neutropenia grade increased after the second cycle compared to baseline *p*-value < 0.05) (Table 14).

During the AC chemotherapy cycles, patients carrying the GG genotype of the *ABCB1* gene exhibited a significant increase in neutropenia grade after the second and fourth cycles, while lymphocytopenia grade significantly increased after the third and fourth cycles compared to baseline (*p* < 0.05). Similarly, among patients with the GA+AA genotype of the *ABCB1* gene, the neutropenia grade showed a significant increase following the second cycle, and the lymphocytopenia grade significantly increased after the fourth cycle compared to pre-treatment levels (*p*-value < 0.05) (Table 14).

##### GIT Toxicity Among the Same Genotype Group Data Analysis Using Two-Way with One-Repeated-Measure ANOVA Test Results

Quantitative gastrointestinal toxicity variables, including nausea, vomiting, diarrhea, constipation, mucositis, and stomachache grades, were analyzed for their correlation with different genotypes using a two-way analysis of variance (ANOVA) under the fit general linear model.

Among patients carrying the CC genotype of the *CYP2C19* gene, the mean grades of vomiting and mucositis significantly increased after each chemotherapy (AC regimen) cycle (*p* < 0.05). Additionally, nausea severity significantly increased after cycles one and two, while constipation severity significantly increased after cycle two compared to pre-treatment levels (point A) (*p*-value < 0.05). Similarly, among patients with the CT+TT genotype of the *CYP2C19* gene, nausea severity was significantly higher after cycles one and two compared to before chemotherapy (*p*-value < 0.05) (Table 15).

For the *ALDH3A1* gene, patients carrying the GG genotype experienced a significant increase in constipation severity after each chemotherapy cycle, nausea severity significantly increased after cycles one and two, vomiting severity significantly increased after cycles one, two, and four, and stomachache severity significantly increased after cycle one compared to baseline (point A) (*p*-value < 0.05). Patients with the GC+CC genotype exhibited significantly higher nausea severity after each chemotherapy cycle, while vomiting severity increased after cycles one and two compared to before chemotherapy (*p*-value < 0.05) (Table 15).

Regarding the *SLC22A16* gene, nausea and vomiting severity significantly increased after cycles one and two, mucositis severity significantly increased after cycles one and three, and stomachache severity significantly increased after cycle one in patients carrying the AA genotype compared to before chemotherapy (*p* < 0.05). In the GG+GA genotype group, nausea and constipation severity significantly increased after cycles one and two, while vomiting severity increased after each chemotherapy cycle compared to pre-treatment levels (*p*-value < 0.05) (Table 15).

For the *ABCB1* gene, patients carrying the GG genotype showed a significant increase in nausea severity after cycles one, two, and three, while vomiting severity increased after cycle one compared to before chemotherapy (*p*-value < 0.05). Among patients with the GA+AA genotype, nausea and mucositis severity significantly increased after cycles one and two, while vomiting severity increased after cycles one, two, and three compared to the baseline (*p*-value < 0.05) (Table 15).

##### Miscellaneous Toxicities Among the Same Genotype Group Data Analysis Using Two-Way with One-Repeated-Measure ANOVA Test Results

Quantitative toxicity variables, including fever, fatigue, amenorrhea, alopecia, headache, skin toxicity, and peripheral neuropathy grades, were analyzed for their correlation with different genotypes using a two-way analysis of variance (ANOVA) under the fit general linear model.

Among patients carrying the CC genotype of the *CYP2C19* gene, fatigue severity significantly increased after cycles one and two, while amenorrhea and skin toxicity severity significantly increased after cycle one. Additionally, alopecia severity significantly increased after all chemotherapy cycles compared to pre-treatment levels (point A) (*p* < 0.05). In patients with the CT+TT genotype of the *CYP2C19* gene, fatigue severity significantly increased after cycle one, and alopecia severity increased after all chemotherapy cycles compared to baseline (*p*-value < 0.05) (Table 16).

For the *ALDH3A1* gene, patients with the GG genotype experienced a significant increase in fatigue severity after cycles one and two, while amenorrhea and fever severity significantly increased after cycle one. Alopecia severity also increased after all chemotherapy cycles compared to pre-treatment levels (*p* < 0.05). Among patients carrying the GC+CC genotype of the *ALDH3A1* gene, peripheral neuropathy severity significantly increased after cycle two, skin toxicity severity increased after cycle one, and alopecia severity increased after all chemotherapy cycles compared to baseline (*p*-value < 0.05) (Table 16).

Regarding the *SLC22A16* gene, patients carrying the GG+GA genotype exhibited a significant increase in fatigue and skin toxicity severity after cycle one compared to before chemotherapy (*p* < 0.05). In the AA genotype group, fatigue severity increased significantly after cycles one and two. Alopecia severity significantly increased throughout all chemotherapy cycles in patients carrying the AA, GG, and GA genotypes compared to pre-treatment levels (*p*-value < 0.05) (Table 16). For the *ABCB1* gene, fatigue severity significantly increased after cycles one and two, while alopecia severity increased after all chemotherapy cycles among patients carrying the GG genotype (*p* < 0.05). In patients with the GA+AA genotype, fatigue, and skin toxicity severity significantly increased after cycle one, and alopecia severity increased after all cycles compared to pre-treatment levels (*p*-value < 0.05) (Table 16).

## 3. Discussion

Pharmacogenetics aims to establish screening tools to optimize pharmacological therapy. This work aimed to identify pharmacogenetic polymorphism that may predict hematological, GIT, and other toxicities in BC patients receiving the AC regimen. Our findings demonstrate that the AC regimen-related chemotoxicity is influenced by a polymorphism in genes involved in drug metabolism (*CYP2C19* and *ALDH3A1*) and drug transport (*ABCB1* and *SLC22A16*).

For the *CYP2C19* gene (rs12248560), the C allele was the predominant homozygous variant, observed in 66.67% of individuals, while the T allele (CT and TT genotypes) represented the minor variant. The *ALDH3A1* gene (rs2228100) had the G allele as the most frequent homozygous variant (47.92%), whereas the C allele (GC and CC genotypes) was less common. For the *SLC22A16* gene (rs6907567), 48.96% of patients carried the AA genotype, while the GA and GG genotypes were less prevalent. The G allele of *ABCB1* gene (rs1045642) was the wild type (GG genotype in 51.04% of participants), while the AA genotype and the GA genotype were the minor variants.

The frequencies of genetic variants are associated with the subject’s ethnic group [70,71,72], which is based on environmental factors and epigenetics [30].

No significant correlation was found between *CYP2C19*, *ALDH3A1*, *SLC22A16*, or *ABCB1* genotypes and age, body surface area, number of positive lymph nodes, and number of lymph nodes removed (*p*-value > 0.05). However, the GG genotype of *ABCB1* was significantly more frequent in married participants.

The CYP2C19*17 variant (rs12248560) contributed to the ultra-rapid metabolism of the *CYP2C19* enzyme substrates [53], including cyclophosphamide [31]. Our study revealed that the CYP2C19*17 variant had the CC as the homozygote major genotype, in line with previous Polish work [31]. Our study found that the CC genotype was linked to an increased recurrence of neutropenia, vomiting, mucositis, and fatigue. According to previous work, the polymorphisms in the CYP450 metabolizing genes had a significant influence on hematological and gastrointestinal toxicity [31]. In addition, higher-grade leukopenia occurred after taking the AC regimen cycles in CC carriers due to slower cyclophosphamide metabolism, consistent with Tecza et al. [31]. Increased lymphocytopenia, amenorrhea, constipation, and skin toxicity grades were also observed in CC carriers. On the other hand, TT and CT genotypes (associated with increased CYP2C19 enzymatic activity) showed lower toxicity risks due to faster cyclophosphamide deactivation [73,74,75].

The GG genotype of *ALDH3A1* was predominant (47.92%), aligning with findings from Afsar et al. and Berdyński et al. [58,76]. The G allele was first described as common and benign, after being detected in subjects with Sjögren–Larsson syndrome [77]. The GG genotypes was associated with a higher risk of fever and recurrent fatigue, while recurrent neutropenia and constipation occurred exclusively in GG carriers.

Anemia severity increased significantly after AC treatment in GG genotype carriers, consistent with previous research by Tecza et al. [31]. Moreover, in the groups that did not carry the G allele, they found no cases of recurrent anemia [31], in line with our findings. This can be explained by the significant role *ALDH3A1* plays in the metabolism of aldophosphamide (one of the cyclophosphamide metabolites) to carboxyphosphamide, influencing its hematological and non-hematological chemotoxicity [78]. The slower rate of detoxification of cyclophosphamide and its accumulation inside the cells may be associated with the presence of the GG genotype of the *ALDH3A1* gene [31].

On the other hand, the GC and CC genotypes exert a significantly higher clearance of cyclophosphamide due to increased *ALDH3A1* expression [79,80].

Tecza et al. reported that the common homozygote allele G of polymorphism (rs2228100; *2) of the *ALDH3A1* gene present in the pathway of the cyclophosphamide metabolism affected leukopenia risks due to weaker detoxification [31]. However, we found no correlation between leukopenia occurrence and the presence of different allele groups of the *ALDH3A1* gene.

On the other hand, the GC and CC genotypes of rs2228100 of the *ALDH3A1* gene showed a significantly higher risk for peripheral neuropathy, recurrent nausea, and stomachache. Also, recurrent thrombocytopenia, alopecia, and vomiting were observed in GG and GC+CC groups. Previous research did not find a direct effect of *ALDH3A1* on cyclophosphamide clearance [81].

Our findings highlight the influence of the *SLC22A16* importer gene on the AC regimen tolerance and efficacy as it regulates intracellular drug concentrations [31]. *SlC22A16* (rs6907567) is an SNP where A is the wild allele and G is the minor one [25,65], in compliance with our findings. The expression of the *SLC22A16* gene is limited to the hematopoietic cells of the bone marrow, which can be explained by its role in hematopoiesis [61]. However, when overexpressed, inflow and susceptibility to the side effects of doxorubicin in the bone marrow increases [31], which may result in dose delay [25]. The AA genotype was associated with recurrent neutropenia, mucositis, and fatigue due to altered drug transport, similar to prior study by Tecza et al. [31]. It was hypothesized that early toxicity occurring before reaching a certain concentration threshold of the cytotoxic drug can occur due to changes in its import and export transporters before the metabolic enzymes start their actions [31]. The polymorphic G allele of the *SLC22A16* gene was reported previously to influence gastrointestinal toxicities, including nausea [31]. However, no significant difference was found between both genotype groups in nausea occurrence. On the other hand, constipation was recurrent in the AG and GG genotypes only.

This study investigated the effect of the common polymorphisms in *ABCB1* (rs1045642), which is an efflux transporter gene, highly expressed in the GIT, bile ducts, kidneys, and blood–tissue barriers. It protects against drugs and endogenous molecules [82]. An association of *ABCB1* with the incidence of anthracyclines toxicity was reported previously [25], especially neutropenia [83,84]. While we found no significant genotype-related differences in neutropenia and lymphocytopenia incidence, recurrent neutropenia and thrombocytopenia were observed only in the GG genotype carriers. The expression of the *ABCB1* efflux transporter is high in the hematopoietic and lymphocyte cells [85]. The myelocyte efflux of doxorubicin may be impaired by having the G allele of *ABCB1* (rs1045642), increasing its hematological toxicity.

Likewise, previous research aimed to assess the use of *ABCB1* polymorphism as a predictor of neutropenia with amrubicin, which is one of the anthracyclines. They found that carriers of the homozygous common type (GG) of *ABCB1* (rs1045642) were at higher risk of developing severe amrubicin-induced neutropenia. The results indicated that *ABCB1* SNP might be a good predictor of worse AC regimen-induced neutropenia [84]. Conversely, there was no influence of the *ABCB1* genetic polymorphism on hematological toxicity in previous research [41]. Polymorphic variants in the efflux transporter gene *ABCB1* may predict fluorouracil, adriamycin, and cytoxan (FAC) regimen’s myelotoxicity, except for leukopenia [31]. Tecza et al. linked *ABCB1* polymorphisms (p.Ile1145=, C allele) to recurrent anemia due to slower drug clearance [31]. However, our study found no significant difference between the *ABCB1* genotypes, concerning anemia occurrence.

Hair follicles express *ABCB1* transporter proteins, which protect against chemotherapy-induced alopecia by reducing its accumulation in the hair follicle epithelium [86,87]. However, although recurrent alopecia occurred in all genotype groups of the *ABCB1* gene, no significant difference was found between *ABCB1* genotypes and its occurrence. Recurrent mucositis and vomiting only occurred in the AA and the GA genotype carriers.

Factors that could justify that some of this study’s results differ from the aforementioned studies are the differences in ethnicity, sample size, and cancer characteristics [72,88]. Studying genetic polymorphisms in transporter and metabolizing genes is a reliable pharmacological tool that can be utilized to understand differences in chemotoxicity. The current study presents evidence of the importance of assessing gene polymorphisms that influence chemotherapy. Particularly, we found that certain functional alleles are significantly associated with AC chemotoxicity in BC patients. This can be used in the future as a screening tool for subjects carrying at-risk genotypes, paving the road for precision medicine and providing a rational use of resources [89,90].

Nonetheless, further pretreatment could be employed at high risk for chemotoxicity patients. Patients at increased risk of AC therapy-induced myelosuppression may be pre-treated with iron, vitamin B6, folic acid, erythropoiesis-stimulating drugs, or colony-stimulating factors, based on their genotype. Those at higher risk for gastrointestinal toxicity may need a modified antiemetic regimen and a modified diet [31].

The present study has several strengths. To date, our research is the first to explore the effect of the SNPs in the *CYP2C19* (rs12248560), *ALDH3A1* (rs2228100), *ABCB1* (rs1045642), and *SLC22A16* (rs6907567) genes on the AC regimen toxicity in Egyptian female patients. Unlike most previous research that focused on a single toxicity type, our study considered several types of toxicities (hematological, gastrointestinal, and miscellaneous). Moreover, we analyzed a prospective cohort. However, this study has some limitations.

First, the small cohort size may limit our ability to detect true associations. Therefore, studying larger cohorts in the future may provide more robust evidence for these associations, facilitating translational research based on the efficacy of the genotype-guided individualization of BC patients. However, the cost-effectiveness of testing large population samples is still questionable. So, this potential benefit should be weighed against the genotyping patients to detect those carrying rare variants of interest. Second, we analyzed only four genes involved in the AC regimen pharmacokinetic pathway, which does not completely cover all the important genes in the transport and metabolism pathways. Finally, our study was carried on Egyptian female BC patients only. Future research should examine genetic variations in male patients and more ethnically diverse groups.

## 4. Materials and Methods

### 4.1. Setting

National Cancer Institute and Al-Hussain University Hospital, Cairo, Egypt.

### 4.2. Study Design

This research follows a prospective cohort study design.

### 4.3. Inclusion Criteria

The study included female breast cancer patients aged 18 to 69 years who were receiving the doxorubicin and cyclophosphamide (AC) chemotherapy regimen. The AC regimen consisted of intravenous administration of 60 mg/m^2^ of doxorubicin (Adriamycin, Pfizer, New York, NY, USA) and 600 mg/m^2^ of cyclophosphamide (Endoxan, Baxter, Halle, Germany) on the first day of a 21-day cycle, repeated for four cycles.

### 4.4. Exclusion Criteria

Patients with any other types of malignancies, previously treated for metastatic disease, with any bone marrow disease, serious cardiac disease, uncontrolled medical conditions, pregnancy, or breastfeeding females were excluded.

### 4.5. Study Procedure and Endpoints

#### 4.5.1. Demographic and Clinical Data

Demographic and clinical data were collected from patient records. Patient data were extracted from medical records and included age, body surface area, marital status, menopausal status, history of oral contraception use, family history, smoking history, affected breast, tumor staging, lymph node involvement, receptor status, histopathological classification, age at diagnosis, type of surgery, chemotherapy setting (neoadjuvant/adjuvant), and number of treatment cycles.

#### 4.5.2. Adverse Events Reporting

Adverse events that occurred at any time point during therapy were reported in accordance with the Common Terminology Criteria for Adverse Events (CTCAEs) [91]. The reported toxicity of the AC regimen included the following:

(a) Hematological toxicities included thrombocytopenia, leukopenia, neutropenia, lymphocytopenia, and anemia. These were assessed through using complete blood count analysis using venous blood samples and were collected in tubes coated with the corresponding amount of ethylenediamine-tetra acetic acid (EDTA). Blood films were stained with Giemsa stain and analyzed before treatment initiation and after each chemotherapy cycle using a Sysmex XN-550 hematology analyzer [92].
(b) Non-hematological toxicities: Data were collected from patient records and telephone follow-ups. Non-hematological toxicities included gastrointestinal (GIT) symptoms such as nausea, vomiting, diarrhea, constipation, mucositis, and stomachache, as well as other miscellaneous toxicities such as fever, fatigue, amenorrhea, alopecia, headache, skin toxicity, and peripheral neuropathy.

#### 4.5.3. Sampling and Genotyping

Venous blood samples (5 mL) were withdrawn from each participant. Baseline hematological parameters, including absolute neutrophil count, absolute lymphocyte count, platelet count, liver and kidney function tests, were recorded to assess toxicity at baseline and after each chemotherapy cycle. DNA was extracted from venous blood samples, using Gene JET Whole Blood Genomic DNA Purification Mini Kit (Thermo Fisher Scientific, Vilnius, Lithuania). Then, the extracted DNA concentration was measured by a Nanodrop-2000 (Thermo Scientific) by measuring the optical density at a wave-length of 260 nm to check the purification of the samples. The genotyping of *CYP2C19*, *ALDH3A1*, *ABCB1*, and *SLC22A16* was performed using real-time PCR (Stepone plus Real-Time PCR, Applied Biosystem, Foster City, CA, USA).

Analysis of data were based on the TaqMan^®^ MGB Probes for allelic discrimination, following the manufacturer’s instructions. Genotyping data were analyzed using TaqMan^®^ Genotyper™ Software (version V1.7.1).

### 4.6. Statistical Analysis

Data were cleaned and analyzed using Excel 365, Minitab 20, and IBM SPSS 26. Quantitative variables included age, body surface area, number of positive lymph nodes, and number of lymph nodes removed. Qualitative variables, including marital status, menopausal status, oral contraception use, family history of BC, smoking history, type of surgery, affected breast, tumor staging (T and N stage), histopathological classification, hormone receptor status (estrogen receptor, progesterone receptor, or human epidermal growth factor receptor 2), and chemotherapy setting (adjuvant/neoadjuvant). Descriptive statistics, including the mean and standard error of the mean were calculated for each genotype. The count and percentage were calculated for the qualitative variables of hormonal status concerning each genotype. Inferential statistics were used to compare between different groups. Box–Cox transformation was used for non-normally distributed variables, following the optimal lambda (λ) method. Data transformations were applied where necessary to meet parametric assumptions. Model fit was assessed using normal residual probability plots.

Qualitative variables (thrombocytopenia, thrombocytosis, leucopenia, leukocytosis, neutropenia, lymphocytopenia, lymphocytosis, anemia, fever, fatigue, nausea, vomiting, diarrhea, constipation, mucositis, amenorrhea, alopecia, headache, skin toxicity, stomachache, and peripheral neuropathy) were analyzed for association with different alleles of *CYP2C19*, *ALDH3A1*, *SLC22A16*, and *ABCB1* genes and tested using Chi-square (X^2^) test.

Quantitative variables (age, body surface area, number of positive lymph nodes, number of lymph nodes removed, hemoglobin, platelets, absolute neutrophil count, white blood cell (WBC) count, absolute lymphocyte count, thrombocytopenia, thrombocytosis, leucopenia, leukocytosis, neutropenia, lymphocytopenia, lymphocytosis, anemia, fever, fatigue, nausea, vomiting, diarrhea, constipation, mucositis, amenorrhea, alopecia, headache, skin toxicity, stomachache, and peripheral neuropathy) correlation with different alleles were tested using different tests including Student’s *t*-test, one-way ANOVA and two-way analysis of variance (ANOVA) under fit general linear model, with one-repeated-measure analysis where five-time points were included for each side effect (before treatment initiation and after each of the four treatment cycles).

Results showed a good fit for different models. The normal residual probability plots showed a linear attitude for all analyses after the transformation of the data. The *p*-value was considered significant at *p* < 0.05. Post hoc analyses of the interactions among all groups were performed using the Tukey test for pairs comparison. The results of the post hoc analyses were represented as letters where groups with the same letters were non-significantly different, while different letters meant significant variation among different groups. Interval plots of quantitative variables showing the mean and median of each variable concerning different genotypes with a 95% confidence interval were generated using Minitab 20.

## 5. Conclusions

In conclusion, this study identified a significant correlation between the SNP variations of the *ALDH3A1* (rs2228100) gene, which plays a role in drug metabolism, and AC regimen-related toxicities in female BC patients. Although the SNPs in *CYP2C19* (rs12248560), *ABCB1* (rs1045642), and *SLC22A16* (rs6907567) genes did not show significant differences between variant alleles in the occurrence of toxicities from the AC regimen, certain genotypes showed a significant influence on the recurrence of certain adverse effects. Given the modest impact linked to each genetic variant identified in our study, future research utilizing multifactorial polymorphic models in larger cohorts is necessary to validate these findings. Such studies may offer valuable insights for personalizing breast cancer treatments, ensuring that genetic screening can be incorporated into clinical practice. The results of this study may provide a rapid, high-accuracy pre-treatment test that helps to spare patients who are likely to exhibit low tolerance to AC treatment, hence increasing the likelihood of completing chemotherapy without delays or morbidity and preserving quality of life by prescribing an alternative treatment.

## Figures and Tables

**Table 1:** Patients’ characteristics.

Gene type		(a) Metabolizing enzyme genes						(b) Transporting enzyme genes					
Gene		*CYP2C19*			*ALDH3A1*			*SLC22A16*			*ABCB1*		
Parameter		CC	CT+TT	*p*-value	GC+CC	GG	*p*-value	AA	GG+GA	*p*-value	AA+GA	GG	*p*-value
Age (year)		47.2 ± 9.88	45 ± 11.33	NS	46.94 ± 10.63	45.96 ± 10.2	NS	44.94 ± 11.66	47.94 ± 8.86	NS	45.94 ± 1.5	47.02 ± 1.51	NS
BSA (m^2^)		1.86 ± 0.15	1.82 ± 0.19	NS	1.86 ± 0.16	1.83 ± 0.16	NS	1.83 ± 0.16	1.86 ± 0.16	NS	1.85 ± 0.02	1.84 ± 0.02	NS
No. of lymph nodes removed		13.12 ± 7.89	14.28 ± 7.07	NS	14.4 ± 8.24	12.64 ± 6.93	NS	13.27 ± 7.8	13.74 ± 7.48	NS	13.6 ± 1.31	13.4 ± 0.98	NS
No. of +ve lymph nodes		2.42 ± 3.27	3.34 ± 6.22	NS	2.91 ± 5.34	2.91 ± 5.34	NS	2.8 ± 3.55	2.64 ± 5.23	NS	2.64 ± 0.55	2.8 ± 0.76	NS
Marital status	Missed	1 (2)	0 (0)	0.36	0 (0)	1 (2)	0.66	1 (2)	0(0)	0.22	1 (2)	0(0)	0.039 *
	Married	52(81)	30(94)		42(84)	40(87)		42(89)	40(82)		37(75.5)	45(95.7)	
	Single	3(5)	0(0)		2(4)	1(2)		0(0)	3(6)		3(6.1)	0(0)	
	Was married	8(13)	2(6)		6(12)	4(9)		4(9)	6(12)		8(16.3)	2(4.3)	
Menopausal status	Postmenopausal	24(38)	13(41)	0.77	21(42)	16(35)	0.47	15(32)	22(45)	0.19	17(34.7)	20(42.6)	0.429
	Premenopausal	40(63)	19(59)		29(58)	30(65)		32(68)	27(55)		32(65.3)	27(57.4)	
Oral contraception	Missed	4(6)	4(13)	0.38	4(8)	4(9)	0.99	3(6)	5(10)	0.73	5(10.2)	3(6.4)	0.726
	No	32(50)	12(38)		23(46)	21(46)		21(45)	23(47)		21(42.9)	23(48.9)	
	Yes	28(44)	16(50)		23(46)	21(46)		23(49)	21(43)		23(46.9)	21(44.7)	
Family history	Missed	3(5)	4(13)	0.22	4(8)	3(7)	0.51	2(4)	5(10)	0.5	5(10.2)	2(4.3)	
	No	44(69)	17(53)		34(68)	27(59)		30(64)	31(63)		32(65.3)	29(61.7)	0.375
	Yes	17(27)	11(34)		12(24)	16(35)		15(32)	13(27)		12(24.5)	16(34)	
Smoking history	No	63(98)	32(100)	0.48	49(98)	46(100)	0.34	46(98)	49(100)	0.31	49(100)	46(97.9)	0.305
	Yes	1(2)	0(0)		1(2)	0(0)		1(2)	0(0)		0(0)	1(2.1)	
Type of surgery	Missed	0(0)	1(3)	0.17	1(2)	0(0)	0.4	0(0)	1(2)	0.81	1(2.04)	0(0)	0.599
	BCS	6(9)	2(6)		3(6)	5(11)		4(9)	4(8)		3(6.12)	5(10.64)	
	MRM	49(77)	28(88)		39(78)	38(83)		38(81)	39(80)		39(79.59)	38(80.85)	
	No surgery	9(14)	1(3)		7(14)	3(7)		5(11)	5(10)		6(12.24)	4(8.51)	
Breast affected	Bilateral	1(2)	0(0)	0.36	1(2)	0(0)	0.33	0(0)	1(2)	0.19	1(2.04)	0(0)	0.477
	Left	36(56)	14(44)		23(46)	27(59)		21(45)	29(59)		27(55.1)	23(48.94)	
	Right	27(42)	18(56)		26(52)	19(41)		26(55)	19(39)		21(42.86)	24(51.06)	
Grade	1	1(0)	0(0)	0.46	0(0)	1(0)	0.47	0(0)	1(0)	0.53	1(2.04)	0(0)	0.401
	2	47(0)	22(0)		36(0)	33(0)		33(0)	36(0)		37(75.51)	32(69.57)	
	3	16(0)	8(0)		14(0)	10(0)		13(0)	11(0)		10(20.41)	14(30.43)	
	4	0(0)	1(0)		0(0)	1(0)		0(0)	1(0)		1(2.04)	0(0)	
Stage-T	1	2(0)	3(0)	0.33	1(0)	4(0)	0.1	4(0)	1(0)	0.26	1(2.33)	4(9.3)	0.165
	2	27(0)	10(0)		15(0)	22(0)		20(0)	17(0)		16(37.21)	21(48.84)	
	3	15(0)	4(0)		12(0)	7(0)		10(0)	9(0)		13(30.23)	6(13.95)	
	4	16(0)	9 (0)		16 (0)	9 (0)		9 (0)	16(0)		13(30.23)	12 (27.91)	
Stage-N	0	18(0)	6 (0)	0.19	12 (0)	12(0)	0.19	13 (0)	11(0)	0.76	13(30.23)	11 (25.58)	0.919
	1	28 (0)	18 (0)		26 (0)	20(0)		23 (0)	23(0)		23(53.49)	23 (53.49)	
	2	11(0)	1 (0)		3(0)	9(0)		6 (0)	6(0)		5 (11.63)	7 (16.28)	
	3	3 (0)	1 (0)		3(0)	1(0)		1(0)	3(0)		2(4.65)	2(4.65)	
Adjuvant/Neoadjuvant	Adjuvant	31(48)	14 (44)	0.68	21(42)	24(52)	0.6	21(45)	24(49)	0.84	22 (44.9)	23 (48.94)	0.41
	Neoadjuvant	29 (45)	17(53)		26(52)	20(43)		23 (49)	23(47)		23(46.94)	23 (48.94)	
	Palliative	4 (6)	1(3)		3(6)	2(4)		3(6)	2(4)		4(8.16)	1(2.13)	
Pathological type	Missed	0 (0)	1(3)	0.37	0(0)	1(2)	0.56	0 (0)	1(2)	0.68	1(2.04)	0(0)	0.549
	Invasive duct/lobular carcinoma	1(2)	0(0)		0(0)	1(2)		0(0)	1(2)		1(2.04)	0(0)	
	Invasive cribriform carcinoma	1(2)	0(0)		1(2)	0(0)		1(2)	0(0)		1(2.04)	0(0)	
	Invasive duct carcinoma	58(91)	28 (88)		44(88)	42(91)		42(89)	44(90)		43 (87.76)	43 (91.49)	
	Invasive lobular carcinoma	2(3)	2(6)		3(6)	1(2)		2(4)	2(4)		1(2.04)	3(6.38)	
	Invasive mammary carcinoma	2(3)	0(0)		1(2)	1(2)		1(2)	1(2)		1(2.04)	1(2.13)	
	Invasive micropapillary carcinoma	0(0)	1(3)		1(2)	0(0)		1(2)	0(0)		1(2.04)	0(0)	
ER	Missed	1(2)	2 (6)	0.57	1(2)	2(4)	0.44	2(4)	1(2)	0.64	1(2.04)	2(4.26)	0.692
	Negative	16(25)	8 (25)		15(30)	9(20)		13(28)	11(22)		13(26.53)	11(23.4)	
	Positive	46(72)	22 (69)		33(66)	35(76)		32(68)	36(73)		34(69.39)	34(72.34)	
	Rt−/left+	1(2)	0 (0)		1(2)	0(0)		0 (0)	1(2)		1(2.04)	0(0)	
PR	Missed	1(2)	2 (6)	0.55	1 (2)	2(4)	0.68	2 (4)	1(2)	0.44	1(2.04)	2 (4.26)	0.853
	Negative	2(3)	0 (0)		1(2)	1(2)		0 (0)	2(4)		1(2.04)	1(2.13)	
	Negative	17(27)	8 (25)		11 (22)	14(30)		11(23)	14(29)		13(26.53)	12 (25.53)	
	Positive	43(67)	22(69)		36(72)	29(63)		34 (72)	31(63)		33(67.35)	32 (68.09)	
	Rt−/left+	1(2)	0(0)		1(2)	0(0)		0(0)	1(2)		1(2.04)	0(0)	
HER2	Missed	1(2)	2(6)	0.53	1(2)	2(4)	0.62	2(4)	1(2)	0.44	1(2.04)	2(4.26)	0.727
	Equivocal	3(5)	1(3)		3(6)	1(2)		3(6)	1(2)		3(6.12)	1(2.13)	
	Negative	44(69)	19(59)		31(62)	32(70)		32 (68)	31(63)		32(65.31)	31(65.96)	
	Positive	16(25)	10(31)		15 (30)	11(24)		10 (21)	16(33)		13(26.53)	13(27.66)	

Data expressed as mean ± standard deviation (SD) or n (%). BCS: Breast-conserving surgery. BSA: body surface area. ER: estrogen receptor. HER2: human epidermal growth factor receptor. MRM: Modified radical mastectomy. No significant difference between the genotype groups (*p*-value > 0.05). PR: progesterone receptor. Stage-N: whether the tumor spread to the lymph node. Stage-T: size of tumor. *: means statistically significant.

**Table 2:** Hematological toxicity correlation with the genotype groups of *CYP2C19* gene assessed at different time points.

*CYP2C19*				
Parameter		CC	CT+TT	*p* Value
Thrombocytopenia A	0	63 (100)	32 (100)	NA
Thrombocytopenia B	0	62 (97)	29 (97)	0.96
	1	2 (3)	1 (3)	
Thrombocytopenia C	0	62 (97)	30 (97)	0.98
	1	2 (3)	1 (3)	
Thrombocytopenia D	0	62 (100)	29 (97)	0.15
	1	0 (0)	1 (3)	
Thrombocytopenia E	0	54 (96)	25 (96)	0.68
	1	1 (2)	0 (0)	
	2	1 (2)	1(4)	
Leukopenia A	0	64 (100)	32 (100)	NA
Leukopenia B	0	59 (92)	29 (97)	0.66
	1	4 (6)	1 (3)	
	2	1 (2)	0 (0)	
Leukopenia C	0	54 (86)	30 (97)	0.26
	1	8 (13)	1 (3)	
	2	1 (2)	0 (0)	
Leukopenia D	0	53 (85)	26 (87)	0.84
	1	6 (10)	2 (7)	
	2	3 (5)	2 (7)	
Leukopenia E	0	47 (84)	25 (96)	0.37
	1	5 (9)	0 (0)	
	2	3 (5)	1 (4)	
	4	1 (2)	0 (0)	
Neutropenia A	0	62 (100)	32 (100)	NA
Neutropenia B	0	59 (92)	30 (97)	0.77
	1	1 (2)	0 (0)	
	2	3 (5)	1 (3)	
	3	1 (2)	0 (0)	
Neutropenia C	0	45 (70)	26 (84)	0.26
	1	4 (6)	0 (0)	
	2	3 (5)	0 (0)	
	3	12(19)	5 (16)	
Neutropenia D	0	56(90)	29(97)	0.49
	1	2(3)	0(0)	
	2	4(6)	1(3)	
Neutropenia E	0	44(79)	22(85)	0.5
	1	0(0)	1(4)	
	2	4(7)	1(4)	
	3	1(2)	0(0)	
	4	7 (13)	2(8)	
Lymphocytopenia A	0	64 (100)	32(100)	NA
Lymphocytopenia B	0	57 (90)	27(90)	0.07
	1	6 (10)	1(3)	
	2	0 (0)	2 (7)	
Lymphocytopenia C	0	54 (84)	28(88)	0.9
	1	7 (11)	3 (9)	
	2	2 (3)	1 (3)	
	3	1 (2)	0 (0)	
Lymphocytopenia D	0	48 (77)	20 (67)	0.48
	1	11 (18)	7 (23)	
	2	3 (5)	3 (10)	
Lymphocytopenia E	0	35 (63)	16 (62)	0.9
	1	13 (23)	7 (27)	
	2	6 (11)	3 (12)	
	3	1 (2)	0 (0)	
	4	1 (2)	0 (0)	
Anemia A	0	56 (89)	26 (81)	0.29
	1	3 (5)	5 (16)	
	2	3 (5)	1 (3)	
	3	1 (2)	0 (0)	
Anemia B	0	49 (78)	23 (77)	0.81
	1	8 (13)	5 (17)	
	2	6 (10)	2 (7)	
Anemia C	0	43 (68)	20 (65)	0.87
	1	14 (22)	7(23)	
	2	6 (10)	4 (13)	
Anemia D	0	41(67)	18 (60)	0.4
	1	11(18)	9 (30)	
	2	9 (15)	3 (10)	
Anemia E	0	33 (60)	14(54)	0.41
	1	14 (25)	6 (23)	
	2	6 (11)	6 (23)	
	3	2 (4)	0 (0)	

Data expressed as n (%). NA = non-applicable. Toxicity was assessed at five time points (A, B, C, D, E) among the genotype group data analyses, where A represents the period before administering the chemotherapy, B means after taking the first cycle of chemotherapy, C means after taking the second cycle of chemotherapy, D means after taking the third cycle of chemotherapy, and E means after taking the fourth cycle of chemotherapy. The occurrence of adverse effects was categorized as follows: A score of 0 indicated that the side effect did not appear at any of the five recorded time points before or after treatment. A score of 1 was assigned if the side effect was absent before treatment but emerged during the course of treatment. A score of 2 indicated that the side effect was already present before treatment, regardless of whether it changed after treatment. A score of 3 was used if the side effect was persistent both before and after treatment. Lastly, a score of 4 signified that the side effect was absent before treatment but appeared consistently after each chemotherapy cycle.

**Table 3:** Hematological toxicity correlation with the genotype groups of *ALDH3A1* gene assessed at different time points.

*ALDH3A1*				
Parameter		GC+CC	GG	*p* Value
Thrombocytopenia A	0	50(100)	45(100)	NA
Thrombocytopenia B	0	47(96)	44(98)	0.61
	1	2(4)	1(2)	
Thrombocytopenia C	0	48(98)	44(96)	0.52
	1	1(2)	2(4)	
Thrombocytopenia D	0	48(100)	43(98)	0.29
	1	0 (0)	1 (2)	
Thrombocytopenia E	0	41 (98)	38 (95)	0.22
	1	1 (2)	0 (0)	
	2	0 (0)	2 (5)	
Leukopenia A	0	50 (100)	46 (100)	NA
Leukopenia B	0	46 (94)	42(93)	0.55
	1	3 (6)	2 (4)	
	2	0 (0)	1 (2)	
Leukopenia C	0	44 (90)	40 (89)	0.57
	1	5 (10)	4(9)	
	2	0 (0)	1(2)	
Leukopenia D	0	44 (92)	35 (80)	0.21
	1	3 (6)	5 (11)	
	2	1 (2)	4 (9)	
Leukopenia E	0	39 (93)	33(83)	0.45
	1	2 (5)	3(8)	
	2	1 (2)	3(8)	
	4	0 (0)	1(3)	
Neutropenia A	0	49 (100)	45(100)	NA
Neutropenia B	0	47 (94)	42(93)	0.39
	1	0 (0)	1(2)	
	2	3 (6)	1(2)	
	3	0(0)	1(2)	
Neutropenia C	0	36 (73)	35 (76)	0.62
	1	1 (2)	3 (7)	
	2	2 (4)	1 (2)	
	3	10 (20)	7 (15)	
Neutropenia D	0	46 (96)	39 (89)	0.33
	1	1 (2)	1 (2)	
	2	1 (2)	4 (9)	
Neutropenia E	0	36 (86)	30 (75)	0.45
	1	0(0)	1 (3)	
	2	2(5)	3 (8)	
	3	1 (2)	0 (0)	
	4	3 (7)	6 (15)	
Lymphocytopenia A	0	50 (100)	46 (100)	NA
Lymphocytopenia B	0	45 (94)	39 (87)	0.45
	1	2 (4)	5 (11)	
	2	1 (2)	1 (2)	
Lymphocytopenia C	0	44 (88)	38 (83)	0.66
	1	5(10)	5 (11)	
	2	1 (2)	2 (4)	
	3	0 (0)	1 (2)	
Lymphocytopenia D	0	35 (73)	33 (75)	0.49
	1	11 (23)	7 (16)	
	2	2 (4)	4 (9)	
Lymphocytopenia E	0	28 (67)	23 (58)	0.49
	1	10 (24)	10 (25)	
	2	3 (7)	6 (15)	
	3	1 (2)	0 (0)	
	4	0 (0)	1 (3)	
Anemia A	0	41 (82)	41 (91)	0.43
	1	6(12)	2 (4)	
	2	2 (4)	2 (4)	
	3	1 (2)	0 (0)	
Anemia B	0	40 (82)	32 (73)	0.52
	1	5 (10)	8 (18)	
	2	4 (8)	4 (9)	
Anemia C	0	35 (71)	28 (62)	0.59
	1	10 (20)	11(24)	
	2	4 (8)	6 (13)	
Anemia D	0	35 (73)	24 (56)	0.17
	1	7 (15)	13 (30)	
	2	6 (13)	6 (14)	
Anemia E	0	27 (64)	20 (51)	0.69
	1	9 (21)	11(28)	
	2	5 (12)	7 (18)	
	3	1 (2)	1 (3)	

Data expressed as n (%). NA = non-applicable. Toxicity was assessed at five time points (A, B, C, D, E) among the genotype group data analyses, where A represents the period before administering the chemotherapy, B means after taking the first cycle of chemotherapy, C means after taking the second cycle of chemotherapy, D means after taking the third cycle of chemotherapy, and E means after taking the fourth cycle of chemotherapy. The occurrence of adverse effects was categorized as follows: A score of 0 indicated that the side effect did not appear at any of the five recorded time points before or after treatment. A score of 1 was assigned if the side effect was absent before treatment but emerged during the course of treatment. A score of 2 indicated that the side effect was already present before treatment, regardless of whether it changed after treatment. A score of 3 was used if the side effect was persistent both before and after treatment. Lastly, a score of 4 signified that the side effect was absent before treatment but appeared consistently after each chemotherapy cycle.

**Table 4:** Hematological toxicity correlation with the genotype groups of *SLC22A16* gene assessed at different time points.

*SLC22A16*				
Parameter	Side Effect	AA	GG+GA	*p* Value
Thrombocytopenia A	0	47 (100)	48 (100)	NA
Thrombocytopenia B	0	43(96)	48 (98)	0.51
	1	2 (4)	1(2)	
Thrombocytopenia C	0	46 (98)	46 (96)	0.57
	1	1 (2)	2 (4)	
Thrombocytopenia D	0	44 (98)	47 (100)	0.3
	1	1(2)	0 (0)	
Thrombocytopenia E	0	41 (95)	38 (97)	0.63
	1	1 (2)	0 (0)	
	2	1 (2)	1 (3)	
Leucopenia A	0	47(100)	49 (100)	NA
Leucopenia B	0	42 (93)	46 (94)	0.55
	1	3 (7)	2(4)	
	2	0 (0)	1(2)	
Leucopenia C	0	41(87)	43 (91)	0.36
	1	6 (13)	3 (6)	
	2	0 (0)	1 (2)	
Leucopenia D	0	38(84)	41(87)	0.68
	1	5(11)	3 (6)	
	2	2(4)	3(6)	
Leucopenia E	0	41(95)	31(79)	0.17
	1	1(2)	4 (10)	
	2	1(2)	3 (8)	
	4	0 (0)	1 (3)	
Neutropenic A	0	46 (100)	48 (100)	NA
Neutropenia B	0	43 (93)	46 (94)	0.39
	1	0 (0)	1 (2)	
	2	3 (7)	1(2)	
	3	0 (0)	1 (2)	
Neutropenia C	0	35 (74)	36(75)	0.32
	1	2 (4)	2 (4)	
	2	0 (0)	3 (6)	
	3	10 (21)	7(15)	
Neutropenia D	0	41(91)	44 (94)	0.32
	1	2 (4)	0 (0)	
	2	2 (4)	3 (6)	
Neutropenia E	0	33(77)	33 (85)	0.56
	1	1 (2)	0 (0)	
	2	3 (7)	2 (5)	
	3	0 (0)	1 (3)	
	4	6 (14)	3 (8)	
Lymphocytopenia A	0	47 (100)	49 (100)	NA
Lymphocytopenia B	0	39 (87)	45 (94)	0.45
	1	5 (11)	2 (4)	
	2	1 (2)	1 (2)	
Lymphocytopenia C	0	39 (83)	43 (88)	0.6
	1	6 (13)	4 (8)	
	2	2 (4)	1 (2)	
	3	0 (0)	1(2)	
Lymphocytopenia D	0	35 (78)	33(70)	0.64
	1	7(16)	11 (23)	
	2	3 (7)	3 (6)	
Lymphocytopenia E	0	27(63)	24 (62)	0.23
	1	8 (19)	12 (31)	
	2	7 (16)	2 (5)	
	3	1 (2)	0 (0)	
	4	0 (0)	1 (3)	
Anemia A	0	42 (89)	40 (83)	0.67
	1	3(6)	5 (10)	
	2	2 (4)	2 (4)	
	3	0 (0)	1 (2)	
Anemia B	0	35 (78)	37 (77)	0.98
	1	6 (13)	7 (15)	
	2	4 (9)	4 (8)	
Anemia C	0	31 (66)	32 (68)	0.66
	1	12 (26)	9 (19)	
	2	4 (9)	6 (13)	
Anemia D	0	29 (64)	30 (65)	1
	1	10 (22)	10(22)	
	2	6 (13)	6 (13)	
Anemia E	0	24 (56)	23 (61)	0.35
	1	11 (26)	9 (24)	
	2	8 (19)	4 (11)	
	3	0 (0)	2 (5)	

Data expressed as n (%). NA = non-applicable. Toxicity was assessed at five time points (A, B, C, D, E) among the genotype group data analyses, where A represents the period before administering the chemotherapy, B means after taking the first cycle of chemotherapy, C means after taking the second cycle of chemotherapy, D means after taking the third cycle of chemotherapy, and E means after taking the fourth cycle of chemotherapy. The occurrence of adverse effects was categorized as follows: A score of 0 indicated that the side effect did not appear at any of the five recorded time points before or after treatment. A score of 1 was assigned if the side effect was absent before treatment but emerged during the course of treatment. A score of 2 indicated that the side effect was already present before treatment, regardless of whether it changed after treatment. A score of 3 was used if the side effect was persistent both before and after treatment. Lastly, a score of 4 signified that the side effect was absent before treatment but appeared consistently after each chemotherapy cycle.

**Table 5:** Hematological toxicity correlation with the genotype groups of *ABCB1* gene assessed at different time points.

*ABCB1*				
		AA+GA	GG	*p*-value
Thrombocytopenia A	0	48 (100)	47 (100)	NA
Thrombocytopenia B	0	48 (97.96)	43 (95.56)	0.508
	1	1 (2.04)	2(4.44)	
Thrombocytopenia C	0	47 (97.92)	45 (95.74)	0.545
	1	1 (2.08)	2(4.26)	
Thrombocytopenia D	0	46 (100)	45 (97.83)	0.315
	1	0 (0)	1 (2.17)	
Thrombocytopenia E	0	40 (100)	39 (92.86)	0.227
	1	0 (0)	1(2.38)	
	2	0 (0)	2 (4.76)	
Leucopenia A	0	49 (100)	47 (100)	NA
leucopenia B	0	47 (95.92)	41(91.11)	0.486
	1	2 (4.08)	3 (6.67)	
	2	0 (0)	1(2.22)	
Leucopenia C	0	46 (95.83)	38 (82.61)	0.105
	1	2 (4.17)	7 (15.22)	
	2	0 (0)	1(2.17)	
Leucopenia D	0	41(89.13)	38 (82.61)	0.384
	1	4 (8.7)	4(8.7)	
	2	1 (2.17)	4 (8.7)	
Leucopenia E	0	35 (87.5)	37 (88.1)	0.751
	1	3 (7.5)	2 (4.76)	
	2	2 (5)	2 (4.76)	
	4	0(0)	1 (2.38)	
Neutropenia A	0	47(100)	47 (100)	NA
Neutropenia B	0	46 (93.88)	43 (93.48)	0.390
	1	1(2.04)	0 (0)	
	2	1(2.04)	3 (6.52)	
	3	1(2.04)	0 (0)	
Neutropenia C	0	37(77.08)	34 (72.34)	0.680
	1	1(2.08)	3 (6.38)	
	2	2 (4.17)	1 (2.13)	
	3	8 (16.67)	9 (19.15)	
Neutropenia D	0	43 (93.48)	42 (91.3)	0.149
	1	2 (4.35)	0 (0)	
	2	1 (2.17)	4 (8.7)	
Neutropenia E	0	33 (82.5)	33 (78.57)	0.533
	1	0 (0)	1 (2.38)	
	2	3 (7.5)	2 (4.76)	
	3	1 (2.5)	0 (0)	
	4	3 (7.5)	6 (14.29)	
Lymphocytopenia A	0	49 (100)	47 (100)	NA
Lymphocytopenia B	0	45 (91.84)	39 (88.64)	0.315
	1	4 (8.16)	3 (6.82)	
	2	0 (0)	2 (4.55)	
Lymphocytopenia C	0	43 (87.76)	39 (82.98)	0.207
	1	6 (12.24)	4 (8.51)	
	2	0 (0)	3 (6.38)	
	3	0 (0)	1 (2.13)	
Lymphocytopenia D	0	38 (82.61)	30 (65.22)	0.106
	1	5 (10.87)	13 (28.26)	
	2	3 (6.52)	3 (6.52)	
Lymphocytopenia E	0	27 (67.5)	24 (57.14)	0.655
	1	9 (22.5)	11 (26.19)	
	2	4 (10)	5 (11.9)	
	3	0 (0)	1 (2.38)	
	4	0 (0)	1 (2.38)	
Anemia A	0	43 (89.58)	39 (82.98)	0.535
	1	4 (8.33)	4 (8.51)	
	2	1 (2.08)	3 (6.38)	
	3	0 (0)	1 (2.13)	
Anemia B	0	36 (75)	36 (80)	0.058
	1	10 (20.83)	3 (6.67)	
	2	2 (4.17)	6 (13.33)	
Anemia C	0	33 (70.21)	30 (63.83)	0.744
	1	10 (21.28)	11 (23.4)	
	2	4 (8.51)	6 (12.77)	
Anemia D	0	33 (73.33)	26 (56.52)	0.134
	1	9 (20)	11 (23.91)	
	2	3 (6.67)	9 (19.57)	
Anemia E	0	24 (61.54)	23 (54.76)	0.328
	1	11 (28.21)	9 (21.43)	
	2	4 (10.26)	8 (19.05)	
	3	0(0)	2(4.76)	

Data expressed as n (%). NA = non-applicable. Toxicity was assessed at five time points (A, B, C, D, E) among the genotype group data analyses, where A represents the period before administering the chemotherapy, B means after taking the first cycle of chemotherapy, C means after taking the second cycle of chemotherapy, D means after taking the third cycle of chemotherapy, and E means after taking the fourth cycle of chemotherapy. The occurrence of adverse effects was categorized as follows: A score of 0 indicated that the side effect did not appear at any of the five recorded time points before or after treatment. A score of 1 was assigned if the side effect was absent before treatment but emerged during the course of treatment. A score of 2 indicated that the side effect was already present before treatment, regardless of whether it changed after treatment. A score of 3 was used if the side effect was persistent both before and after treatment. Lastly, a score of 4 signified that the side effect was absent before treatment but appeared consistently after each chemotherapy cycle.

**Table 6:** GIT toxicity correlation with the genotype groups of *CYP2C19* gene assessed at different time points.

*CYP2C19*				
Parameter		CC	CT+TT	*p* value
Nausea A	0	64 (100)	32 (100)	NA
Nausea B	0	47 (73)	19 (59)	0.16
	1	17 (27)	13 (41)	
Nausea C	1	64 (100)	32 (100)	NA
Nausea D	0	53 (84)	25 (78)	0.47
	1	10 (16)	7 (22)	
Nausea E	0	55 (86)	26 (81)	0.55
	1	9 (14)	6 (19)	
Vomiting A	0	64 (100)	32 (100)	NA
Vomiting B	0	38 (59)	23 (72)	0.53
	1	15 (23)	5 (16)	
	2	9 (14)	4 (13)	
	3	2 (3)	0 (0)	
Vomiting C	0	39 (61)	24 (75)	0.47
	1	15 (23)	4 (13)	
	2	7 (11)	2 (6)	
	3	3 (5)	2 (6)	
Vomiting D	0	41(65)	22 (69)	0.22
	1	18 (29)	5 (16)	
	2	4 (6)	4 (13)	
	3	0 (0)	1 (3)	
Vomiting E	0	41 (65)	24 (75)	0.41
	1	13 (21)	7 (22)	
	2	8 (13)	1 (3)	
	3	1 (2)	0 (0)	
Diarrhea A	0	64 (100)	32 (100)	NA
Diarrhea B	0	57 (89)	28 (88)	0.19
	1	6 (9)	2 (6)	
	2	0 (0)	2 (6)	
	3	1 (2)	0 (0)	
Diarrhea C	0	57 (89)	29 (91)	0.84
	1	5 (8)	2 (6)	
	2	1 (2)	1 (3)	
	3	1 (2)	0 (0)	
Diarrhea D	0	57 (90)	30 (94)	0.74
	1	5 (8)	2 (6)	
	3	1 (2)	0 (0)	
Diarrhea E	0	57 (90)	30 (94)	0.74
	1	5 (8)	2 (6)	
	2	1 (2)	0 (0)	
Constipation A	0	64 (100)	32 (100)	NA
Constipation B	0	51 (80)	24 (75)	0.81
	1	12 (19)	7 (22)	
	2	1 (2)	1 (3)	
Constipation C	0	49 (77)	27 (84)	0.65
	1	13 (20)	4 (13)	
	2	1(2)	1 (3)	
	3	1 (2)	0 (0)	
Constipation D	0	52 (83)	28 (88)	0.36
	1	10(16)	3 (9)	
	2	1(2)	0 (0)	
	3	0 (0)	1 (3)	
Constipation E	0	52 (83)	29 (91)	0.55
	1	9 (14)	2 (6)	
	2	1 (2)	1 (3)	
	3	1 (2)	0 (0)	
Mucositis A	0	64 (100)	32 (100)	NA
Mucositis B	0	49 (77)	27 (87)	0.23
	1	15 (23)	4 (13)	
Mucositis C	0	51 (80)	27 (84)	0.58
	1	13 (20)	5 (16)	
Mucositis D	0	49 (78)	28 (88)	0.25
	1	14 (22)	4 (13)	
Mucositis E	0	50 (79)	30 (94)	0.07
	1	13 (21)	2 (6)	
Stomachache A	0	64 (100)	32 (100)	NA
Stomachache B	0	54 (84)	26 (81)	0.7
	1	10 (16)	6 (19)	
Stomachache C	0	57 (89)	28 (88)	0.82
	1	7(11)	4(13)	
Stomachache D	0	58 (91)	27(84)	0.37
	1	6 (9)	5 (16)	
Stomachache E	0	53 (85)	28 (88)	0.79
	1	9 (15)	4 (13)	

Data expressed as n (%). NA = non-applicable. Toxicity was assessed at five time points (A, B, C, D, E) among the genotype group data analyses, where A represents the period before administering the chemotherapy, B means after taking the first cycle of chemotherapy, C means after taking the second cycle of chemotherapy, D means after taking the third cycle of chemotherapy, and E means after taking the fourth cycle of chemotherapy. The occurrence of adverse effects was categorized as follows: A score of 0 indicated that the side effect did not appear at any of the five recorded time points before or after treatment. A score of 1 was assigned if the side effect was absent before treatment but emerged during the course of treatment. A score of 2 indicated that the side effect was already present before treatment, regardless of whether it changed after treatment. A score of 3 was used if the side effect was persistent both before and after treatment. Lastly, a score of 4 signified that the side effect was absent before treatment but appeared consistently after each chemotherapy cycle.

**Table 7:** GIT toxicity correlation with the genotype groups of *ALDH3A1* gene assessed at different time points.

*ALDH3A1*				
Parameter		GC+CC	GG	*p* value
Nausea A	0	50 (100)	46 (100)	NA
Nausea B	0	33 (66)	33 (72)	0.54
	1	17 (34)	13 (28)	
Nausea C	1	50 (100)	46 (100)	NA
Nausea D	0	36 (73)	42 (91)	0.023 *
	1	13(27)	4 (9)	
Nausea E	0	39 (78)	42 (91)	0.07
	1	11 (22)	4 (9)	
Vomiting A	0	50 (100)	46 (100)	NA
Vomiting B	0	31(62)	30 (65)	0.5
	1	13 (26)	7(15)	
	2	5 (10)	8(17)	
	3	1 (2)	1 (2)	
Vomiting C	0	36 (72)	27(59)	0.18
	1	6 (12)	13 (28)	
	2	6 (12)	3 (7)	
	3	2 (4)	3 (7)	
Vomiting D	0	33 (67)	30 (65)	0.66
	1	12 (24)	11 (24)	
	2	3 (6)	5 (11)	
	3	1(2)	0 (0)	
Vomiting E	0	35 (71)	30 (65)	0.66
	1	9 (18)	11 (24)	
	2	5(10)	4 (9)	
	3	0(0)	1 (2)	
Diarrhea A	0	50(100)	46 (100)	NA
Diarrhea B	0	43(86)	42 (91)	0.42
	1	4(8)	4 (9)	
	2	2 (4)	0 (0)	
	3	1 (2)	0 (0)	
Diarrhea C	0	44 (88)	42 (91)	0.8
	1	4 (8)	3 (7)	
	2	1 (2)	1 (2)	
	3	1 (2)	0 (0)	
Diarrhea D	0	44 (90)	43 (93)	0.59
	1	4 (8)	3 (7)	
	3	1 (2)	0 (0)	
Diarrhea E	0	44 (90)	43 (93)	0.59
	1	4 (8)	3 (7)	
	2	1 (2)	0 (0)	
Constipation A	0	50 (100)	46 (100)	NA
Constipation B	0	43 (86)	32 (70)	0.09
	1	7 (14)	12 (26)	
	2	0 (0)	2 (4)	
Constipation C	0	43 (86)	33 (72)	0.2
	1	7 (14)	10 (22)	
	2	0 (0)	2 (4)	
	3	0 (0)	1 (2)	
Constipation D	0	45 (92)	35 (76)	0.17
	1	4 (8)	9 (20)	
	2	0 (0)	1 (2)	
	3	0 (0)	1 (2)	
Constipation E	0	45 (92)	36 (78)	0.19
	1	4 (8)	7 (15)	
	2	0 (0)	2 (4)	
	3	0 (0)	1 (2)	
Mucositis A	0	50 (100)	46 (100)	NA
Mucositis B	0	40 (82)	36 (78)	0.68
	1	9 (18)	10 (22)	
Mucositis C	0	41(82)	37 (80)	0.84
	1	9(18)	9 (20)	
Mucositis D	0	41(84)	36 (78)	0.5
	1	8(16)	10 (22)	
Mucositis E	0	43(88)	37 (80)	0.33
	1	6(12)	9 (20)	
Stomachache A	0	50(100)	46 (100)	NA
Stomachache B	0	45(90)	35 (76)	0.07
	1	5(10)	11 (24)	
Stomachache C	0	46(92)	39(85)	0.27
	1	4(8)	7 (15)	
Stomachache D	0	41(82)	44 (96)	0.036 *
	1	9(18)	2 (4)	
Stomachache E	0	44(92)	37 (80)	0.11
	1	4(8)	9 (20)	

Data expressed as n (%). *: means statistically significant. NA = non-applicable. Toxicity was assessed at five time points (A, B, C, D, E) among the genotype group data analyses, where A represents the period before administering the chemotherapy, B means after taking the first cycle of chemotherapy, C means after taking the second cycle of chemotherapy, D means after taking the third cycle of chemotherapy, and E means after taking the fourth cycle of chemotherapy. The occurrence of adverse effects was categorized as follows: A score of 0 indicated that the side effect did not appear at any of the five recorded time points before or after treatment. A score of 1 was assigned if the side effect was absent before treatment but emerged during the course of treatment. A score of 2 indicated that the side effect was already present before treatment, regardless of whether it changed after treatment. A score of 3 was used if the side effect was persistent both before and after treatment. Lastly, a score of 4 signified that the side effect was absent before treatment but appeared consistently after each chemotherapy cycle.

**Table 8:** GIT toxicity correlation with the genotype groups of *SLC22A16* gene assessed at different time points.

Parameter	Side Effect	AA	GG+GA	*p* Value
Nausea A	0	47(100)	49(100)	NA
Nausea B	0	32(68)	34(69)	0.89
	1	15(32)	15(31)	
Nausea C	1	47(100)	49(100)	NA
Nausea D	0	38(81)	40(83)	0.75
	1	9(19)	8(17)	
Nausea E	0	40(85)	41(84)	0.85
	1	7(15)	8(16)	
Vomiting A	0	47(100)	49(100)	NA
Vomiting B	0	27(57)	34(69)	0.15
	1	13(28)	7(14)	
	2	5(11)	8(16)	
	3	2(4)	0(0)	
Vomiting C	0	32(68)	31(63)	0.75
	1	10(21)	9(18)	
	2	3(6)	6(12)	
	3	2(4)	3(6)	
Vomiting D	0	32(68)	31(65)	0.67
	1	11(23)	12(25)	
	2	3(6)	5(10)	
	3	1(2)	0(0)	
Vomiting E	0	34(72)	31(65)	0.51
	1	9(19)	11(23)	
	2	3(6)	6(13)	
	3	1(2)	0(0)	
Diarrhea A	0	47(100)	49(100)	NA
Diarrhea B	0	43(91)	42(86)	0.4
	1	4(9)	4(8)	
	2	0(0)	2(4)	
	3	0(0)	1(2)	
Diarrhea C	0	44(94)	42(86)	0.51
	1	2(4)	5(10)	
	2	1(2)	1(2)	
	3	0(0)	1(2)	
Diarrhea D	0	44(94)	43(90)	0.56
	1	3(6)	4(8)	
	3	0(0)	1(2)	
Diarrhea E	0	44(94)	43(90)	0.56
	1	3(6)	4(8)	
	2	0(0)	1(2)	
Constipation A	0	47(100)	49(100)	NA
Constipation B	0	39(83)	36(73)	0.28
	1	8(17)	11(22)	
	2	0(0)	2(4)	
Constipation C	0	38(81)	38(78)	0.39
	1	9(19)	8(16)	
	2	0(0)	2(4)	
	3	0(0)	1(2)	
Constipation D	0	40(85)	40(83)	0.56
	1	7(15)	6(13)	
	2	0(0)	1(2)	
	3	0(0)	1(2)	
Constipation E	0	43(91)	38(79)	0.25
	1	4(9)	7(15)	
	2	0(0)	2(4)	
	3	0(0)	1(2)	
Mucositis A	0	47(100)	49(100)	NA
Mucositis B	0	36(77)	40(83)	0.41
	1	11(23)	8(17)	
Mucositis C	0	37(79)	41(84)	0.53
	1	10(21)	8(16)	
Mucositis D	0	36(77)	41(85)	0.27
	1	11(23)	7(15)	
Mucositis E	0	37(79)	43(90)	0.15
	1	10(21)	5(10)	
Stomachache A	0	47(100)	49(100)	NA
Stomachache B	0	37(79)	43(88)	0.24
	1	10(21)	6(12)	
stomachache C	0	41(87)	44(90)	0.69
	1	6(13)	5(10)	
Stomachache D	0	40(85)	45(92)	0.3
	1	7(15)	4(8)	
Stomachache E	0	38(83)	43(90)	0.33
	1	8(17)	5(10)	

Data expressed as n (%). NA = non-applicable. Toxicity was assessed at five time points (A, B, C, D, E) among the genotype group data analyses, where A represents the period before administering the chemotherapy, B means after taking the first cycle of chemotherapy, C means after taking the second cycle of chemotherapy, D means after taking the third cycle of chemotherapy, and E means after taking the fourth cycle of chemotherapy. The occurrence of adverse effects was categorized as follows: A score of 0 indicated that the side effect did not appear at any of the five recorded time points before or after treatment. A score of 1 was assigned if the side effect was absent before treatment but emerged during the course of treatment. A score of 2 indicated that the side effect was already present before treatment, regardless of whether it changed after treatment. A score of 3 was used if the side effect was persistent both before and after treatment. Lastly, a score of 4 signified that the side effect was absent before treatment but appeared consistently after each chemotherapy cycle.

**Table 9:** GIT toxicity correlation with the genotype groups of *ABCB1* gene assessed at different time points.

Parameter	Side Effect	AA+GA	GG	*p*-Value
Nausea A	0	49(100)	47(100)	NA
Nausea B	0	37(75.5)	29(61.7)	0.145
	1	12(24.5)	18(38.3)	
Nausea C	1	49(100)	47(100)	NA
Nausea D	0	42(85.7)	36(78.3)	0.344
	1	7(14.3)	10(21.7)	
Nausea E	0	43(87.8)	38(80.9)	0.352
	1	6(12.2)	9(19.1)	
Vomiting grade A	0	49(100)	47(100)	NA
Vomiting grade B	0	35(71.4)	26(55.3)	0.207
	1	6(12.2)	14(29.8)	
	2	7(14.3)	6(12.8)	
	3	1(2)	1(2.1)	
Vomiting grade C	0	32(65.3)	31(66)	0.252
	1	7(14.3)	12(25.5)	
	2	6(12.2)	3(6.4)	
	3	4(8.2)	1(2.1)	
Vomiting grade D	0	32(65.3)	31(67.4)	0.345
	1	10(20.4)	13(28.3)	
	2	6(12.2)	2(4.3)	
	3	1(2)	0(0)	
Vomiting grade E	0	36(73.5)	29(63)	0.054
	1	6(12.2)	14(30.4)	
	2	7(14.3)	2(4.3)	
	3	0(0)	1(2.2)	
Diarrhea grade A	0	49(100)	47(100)	
Diarrhea grade B	0	43(87.8)	42(89.4)	0.396
	1	4(8.2)	4(8.5)	
	2	2(4.1)	0(0)	
	3	0(0)	1(2.1)	
Diarrhea grade C	0	42(85.7)	44(93.6)	0.232
	1	5(10.2)	2(4.3)	
	2	2(4.1)	0(0)	
	3	0(0)	1(2.1)	
Diarrhea grade D	0	44(89.8)	43(93.5)	0.332
	1	5(10.2)	2(4.3)	
	3	0(0)	1(2.2)	
Diarrhea grade E	0	44(89.8)	43(93.5)	0.332
	1	5(10.2)	2(4.3)	
	2	0(0)	1(2.2)	
Constipation grade A	0	49(100)	47(100)	
Constipation grade B	0	37(75.5)	38(80.9)	0.800
	1	11(22.4)	8(17)	
	2	1(2)	1(2.1)	
Constipation grade C	0	38(77.6)	38(80.9)	0.797
	1	9(18.4)	8(17)	
	2	1(2)	1(2.1)	
	3	1(2)	0(0)	
Constipation grade D	0	41(83.7)	39(84.8)	0.565
	1	7(14.3)	6(13)	
	2	1(2)	0(0)	
	3	0(0)	1(2.2)	
Constipation grade E	0	42(85.7)	39(84.8)	0.775
	1	5(10.2)	6(13)	
	2	1(2)	1(2.2)	
	3	1(2)	0(0)	
Mucositis A	0	49(100)	47(100)	
Mucositis B	0	37(77.1)	39(83)	0.473
	1	11(22.9)	8(17)	
Mucositis C	0	38(77.6)	40(85.1)	0.343
	1	11(22.4)	7(14.9)	
Mucositis D	0	39(79.6)	38(82.6)	0.708
	1	10(20.4)	8(17.4)	
Mucositis E	0	41(83.7)	39(84.8)	0.882
	1	8(16.3)	7(15.2)	
Stomachache A	0	49(100)	47(100)	
Stomachache B	0	41(83.7)	39(83)	0.927
	1	8(16.3)	8(17)	
Stomachache C	0	44(89.8)	41(87.2)	0.694
	1	5(10.2)	6(12.8)	
Stomachache D	0	44(89.8)	41(87.2)	0.694
	1	5(10.2)	6(12.8)	
Stomachache E	0	42(87.5)	39(84.8)	0.703
	1	6(12.5)	7(15.2)	

Data expressed as n (%). NA = non-applicable. Toxicity was assessed at five time points (A, B, C, D, E) among the genotype group data analyses, where A represents the period before administering the chemotherapy, B means after taking the first cycle of chemotherapy, C means after taking the second cycle of chemotherapy, D means after taking the third cycle of chemotherapy, and E means after taking the fourth cycle of chemotherapy. The occurrence of adverse effects was categorized as follows: A score of 0 indicated that the side effect did not appear at any of the five recorded time points before or after treatment. A score of 1 was assigned if the side effect was absent before treatment but emerged during the course of treatment. A score of 2 indicated that the side effect was already present before treatment, regardless of whether it changed after treatment. A score of 3 was used if the side effect was persistent both before and after treatment. Lastly, a score of 4 signified that the side effect was absent before treatment but appeared consistently after each chemotherapy cycle.

**Table 10:** Miscellaneous toxicity correlation with the genotype groups of *CYP2C19* gene assessed at different time points.

*CYP2C19*				
Parameter		CC	CT+TT	*p* value
Fever A	0	64(100)	32(100)	NA
Fever B	0	58(92)	31(97)	0.36
	1	5(8)	1(3)	
Fever C	0	60(94)	31(97)	0.52
	1	4(6)	1(3)	
Fever D	0	60(95)	30(94)	0.76
	1	3(5)	2(6)	
Fever E	0	62(97)	32(100)	0.31
	1	2(3)	0(0)	
Fatigue A	0	64(100)	32(100)	NA
Fatigue B	0	44(69)	19(59)	0.36
	1	20(31)	13(41)	
Fatigue C	0	50(78)	23(72)	0.5
	1	14(22)	9(28)	
Fatigue D	0	52(83)	24(75)	0.39
	1	11(17)	8(25)	
Fatigue E	0	54(84)	25(78)	0.45
	1	10(16)	7(22)	
Amenorrhea A	0	64(100)	32(100)	NA
Amenorrhea B	0	57(89)	30(94)	0.46
	1	7(11)	2(6)	
Amenorrhea C	0	61(97)	30(94)	0.48
	1	2(3)	2(6)	
Amenorrhea D	0	63(98)	30(94)	0.21
	1	1(2)	2(6)	
Amenorrhea E	0	61(97)	31(97)	0.99
	1	2(3)	1(3)	
Alopecia A	0	64(100)	32(100)	NA
Alopecia B	1	64(100)	32(100)	NA
Alopecia C	1	64(100)	32(100)	NA
Alopecia D	1	64(100)	32(100)	NA
Alopecia E	1	64(100)	32(100)	NA
Headache A	0	64(100)	32(100)	NA
Headache B	0	57(89)	28(88)	0.82
	1	7(11)	4(13)	
Headache C	0	61(95)	30(94)	0.75
	1	3(5)	2(6)	
Headache D	0	61(95)	29(91)	0.37
	1	3(5)	3(9)	
Headache E	0	60(94)	30(94)	1
	1	4(6)	2(6)	
Skin toxicity A	0	64(100)	32(100)	NA
Skin toxicity B	0	51(80)	27(84)	0.58
	1	13(20)	5(16)	
Skin toxicity C	0	59(92)	30(94)	0.78
	1	5(8)	2(6)	
Skin toxicity D	0	55(86)	30(94)	0.26
	1	9(14)	2(6)	
Skin toxicity E	0	55(89)	29(91)	0.78
	1	7(11)	3(9)	
Peripheral neuropathy A	0	64(100)	32(100)	NA
Peripheral neuropathy B	0	60(94)	31(97)	0.52
	1	4(6)	1(3)	
Peripheral neuropathy C	0	58(91)	31(97)	0.27
	1	6(9)	1(3)	
Peripheral neuropathy D	0	60(94)	32(100)	0.15
	1	4(6)	0(0)	
Peripheral neuropathy E	0	58(92)	31(97)	0.36
	1	5(8)	1(3)	

Data expressed as n (%). NA = non-applicable. Toxicity was assessed at five time points (A, B, C, D, E) among the genotype group data analyses, where A represents the period before administering the chemotherapy, B means after taking the first cycle of chemotherapy, C means after taking the second cycle of chemotherapy, D means after taking the third cycle of chemotherapy, and E means after taking the fourth cycle of chemotherapy. The occurrence of adverse effects was categorized as follows: A score of 0 indicated that the side effect did not appear at any of the five recorded time points before or after treatment. A score of 1 was assigned if the side effect was absent before treatment but emerged during the course of treatment. A score of 2 indicated that the side effect was already present before treatment, regardless of whether it changed after treatment. A score of 3 was used if the side effect was persistent both before and after treatment. Lastly, a score of 4 signified that the side effect was absent before treatment but appeared consistently after each chemotherapy cycle.

**Table 11:** Miscellaneous toxicity correlation with the genotype groups of *ALDH3A1* gene assessed at different time points.

*ALDH3A1*				
Parameter		GC+CC	GG	*p* value
Fever A	0	50(100)	46(100)	NA
Fever B	0	49(100)	40(87)	0.009 *
	1	0(0)	6(13)	
Fever C	0	50(100)	41(89)	0.017 *
	1	0(0)	5(11)	
Fever D	0	49(100)	41(89)	0.018 *
	1	0(0)	5(11)	
Fever E	0	50(100)	44(96)	0.14
	1	0(0)	2(4)	
Fatigue A	0	50(100)	46(100)	NA
Fatigue B	0	39(78)	24(52)	0.008 *
	1	11(22)	22(48)	
Fatigue C	0	41(82)	32(70)	0.15
	1	9(18)	14(30)	
Fatigue D	0	41(84)	35(76)	0.36
	1	8(16)	11(24)	
Fatigue E	0	42(84)	37(80)	0.65
	1	8(16)	9(20)	
Amenorrhea A	0	50(100)	46(100)	NA
Amenorrhea B	0	47(94)	40(87)	0.24
	1	3(6)	6(13)	
Amenorrhea C	0	47(96)	44(96)	0.95
	1	2(4)	2(4)	
Amenorrhea D	0	48(96)	45(98)	0.61
	1	2(4)	1(2)	
Amenorrhea E	0	47(96)	45(98)	0.6
	1	2(4)	1(2)	
Alopecia A	0	50(100)	46(100)	NA
Alopecia B	1	50(100)	46(100)	NA
Alopecia C	1	50(100)	46(100)	NA
Alopecia D	1	50(100)	46(100)	NA
Alopecia E	1	50(100)	46(100)	NA
Headache A	0	50(100)	46(100)	NA
Headache B	0	44(88)	41(89)	0.86
	1	6(12)	5(11)	
Headache C	0	48(96)	43(93)	0.58
	1	2(4)	3(7)	
Headache D	0	48(96)	42(91)	0.34
	1	2(4)	4(9)	
Headache E	0	48(96)	42(91)	0.34
	1	2(4)	4(9)	
Skin toxicity A	0	50(100)	46(100)	NA
Skin toxicity B	0	39(78)	39(85)	0.4
	1	11(22)	7(15)	
Skin toxicity C	0	45(90)	44(96)	0.29
	1	5(10)	2(4)	
Skin toxicity D	0	46(92)	39(85)	0.27
	1	4(8)	7(15)	
Skin toxicity E	0	41(84)	43(96)	0.06
	1	8(16)	2(4)	
Peripheral neuropathy A	0	50(100)	46(100)	NA
Peripheral neuropathy B	0	46(92)	45(98)	0.2
	1	4(8)	1(2)	
Peripheral neuropathy C	0	43(86)	46(100)	0.008 *
	1	7(14)	0(0)	
Peripheral neuropathy D	0	48(96)	44(96)	0.93
	1	2(4)	2(4)	
Peripheral neuropathy E	0	46(94)	43(93)	0.94
	1	3(6)	3(7)	

Data expressed as n (%). *: means statistically significant. NA = non-applicable. Toxicity was assessed at five time points (A, B, C, D, E) among the genotype group data analyses, where A represents the period before administering the chemotherapy, B means after taking the first cycle of chemotherapy, C means after taking the second cycle of chemotherapy, D means after taking the third cycle of chemotherapy, and E means after taking the fourth cycle of chemotherapy. The occurrence of adverse effects was categorized as follows: A score of 0 indicated that the side effect did not appear at any of the five recorded time points before or after treatment. A score of 1 was assigned if the side effect was absent before treatment but emerged during the course of treatment. A score of 2 indicated that the side effect was already present before treatment, regardless of whether it changed after treatment. A score of 3 was used if the side effect was persistent both before and after treatment. Lastly, a score of 4 signified that the side effect was absent before treatment but appeared consistently after each chemotherapy cycle.

**Table 12:** Miscellaneous toxicity correlation with the genotype groups of *SLC22A16* gene assessed at different time points.

Parameter	Side Effect	AA	GG+GA	*p* Value
Fever A	0	47(100)	49(100)	NA
Fever B	0	43(91)	46(96)	0.38
	1	4(9)	2(4)	
Fever C	0	45(96)	46(94)	0.68
	1	2(4)	3(6)	
Fever D	0	45(96)	45(94)	0.66
	1	2(4)	3(6)	
Fever E	0	46(98)	48(98)	0.98
	1	1(2)	1(2)	
Fatigue A	0	47(100)	49(100)	NA
Fatigue B	0	31(66)	32(65)	0.95
	1	16(34)	17(35)	
Fatigue C	0	34(72)	39(80)	0.41
	1	13(28)	10(20)	
Fatigue D	0	37(79)	39(81)	0.76
	1	10(21)	9(19)	
Fatigue E	0	39(83)	40(82)	0.86
	1	8(17)	9(18)	
Amenorrhea A	0	47(100)	49(100)	NA
Amenorrhea B	0	43(91)	44(90)	0.78
	1	4(9)	5(10)	
Amenorrhea C	0	46(98)	45(94)	0.32
	1	1(2)	3(6)	
Amenorrhea D	0	46(98)	47(96)	0.58
	1	1(2)	2(4)	
Amenorrhea E	0	46(98)	46(96)	0.57
	1	1(2)	2(4)	
Alopecia A	0	47(100)	49(100)	NA
Alopecia B	1	47(100)	49(100)	NA
Alopecia C	1	47(100)	49(100)	NA
Alopecia D	1	47(100)	49(100)	NA
Alopecia E	1	47(100)	49(100)	NA
Headache A	0	47(100)	49(100)	NA
Headache B	0	40(85)	45(92)	0.3
	1	7(15)	4(8)	
Headache C	0	44(94)	47(96)	0.61
	1	3(6)	2(4)	
Headache D	0	44(94)	46(94)	0.96
	1	3(6)	3(6)	
Headache E	0	43(91)	47(96)	0.37
	1	4(9)	2(4)	
Skin toxicity A	0	47(100)	49(100)	NA
Skin toxicity B	0	39(83)	39(80)	0.67
	1	8(17)	10(20)	
Skin toxicity C	0	43(91)	46(94)	0.65
	1	4(9)	3(6)	
Skin toxicity D	0	42(89)	43(88)	0.8
	1	5(11)	6(12)	
Skin toxicity E	0	43(91)	41(87)	0.5
	1	4(9)	6(13)	
Peripheral neuropathy A	0	47(100)	49(100)	NA
Peripheral neuropathy B	0	44(94)	47(96)	0.61
	1	3(6)	2(4)	
Peripheral neuropathy C	0	44(94)	45(92)	0.74
	1	3(6)	4(8)	
Peripheral neuropathy D	0	44(94)	48(98)	0.29
	1	3(6)	1(2)	
Peripheral neuropathy E	0	45(96)	44(92)	0.41
	1	2(4)	4(8)	

Data expressed as n (%). NA = non-applicable. Toxicity was assessed at five time points (A, B, C, D, E) among the genotype group data analyses, where A represents the period before administering the chemotherapy, B means after taking the first cycle of chemotherapy, C means after taking the second cycle of chemotherapy, D means after taking the third cycle of chemotherapy, and E means after taking the fourth cycle of chemotherapy. The occurrence of adverse effects was categorized as follows: A score of 0 indicated that the side effect did not appear at any of the five recorded time points before or after treatment. A score of 1 was assigned if the side effect was absent before treatment but emerged during the course of treatment. A score of 2 indicated that the side effect was already present before treatment, regardless of whether it changed after treatment. A score of 3 was used if the side effect was persistent both before and after treatment. Lastly, a score of 4 signified that the side effect was absent before treatment but appeared consistently after each chemotherapy cycle.

**Table 13:** Miscellaneous toxicity correlation with the genotype groups of *ABCB1* gene assessed at different time points.

		*ABCB1*		
		AA+GA	GG	*p* value
Fever A	0	49(100)	47(100)	NA
Fever B	0	45(91.8)	44(95.7)	0.445
	1	4(8.2)	2(4.3)	
Fever C	0	47(95.9)	44(93.6)	0.612
	1	2(4.1)	3(6.4)	
Fever D	0	46(93.9)	44(95.7)	0.699
	1	3(6.1)	2(4.3)	
Fever E	0	47(95.9)	47(100)	0.162
	1	2(4.1)	0(0)	
Fatigue A	0	49(100)	47(100)	NA
Fatigue B	0	31(63.3)	32(68.1)	0.619
	1	18(36.7)	15(31.9)	
Fatigue C	0	38(77.6)	35(74.5)	0.724
	1	11(22.4)	12(25.5)	
Fatigue D	0	38(77.6)	38(82.6)	0.538
	1	11(22.4)	8(17.4)	
Fatigue E	0	38(77.6)	41(87.2)	0.214
	1	11(22.4)	6(12.8)	
Amenorrhea A	0	49(100)	47(100)	NA
Amenorrhea B	0	43(87.8)	44(93.6)	0.325
	1	6(12.2)	3(6.4)	
Amenorrhea C	0	47(95.9)	44(95.7)	0.949
	1	2(4.1)	2(4.3)	
Amenorrhea D	0	47(95.9)	46(97.9)	0.582
	1	2(4.1)	1(2.1)	
Amenorrhea E	0	47(95.9)	45(97.8)	0.595
	1	2(4.1)	1(2.2)	
Alopecia A	0	49(100)	47(100)	NA
Alopecia B	1	49(100)	47(100)	NA
Alopecia C	1	49(100)	47(100)	NA
Alopecia D	1	49(100)	47(100)	NA
Alopecia E	1	49(100)	47(100)	NA
Headache A	0	49(100)	47(100)	NA
Headache B	0	43(87.8)	42(89.4)	0.805
	1	6(12.2)	5(10.6)	
Headache C	0	47(95.9)	44(93.6)	0.612
	1	2(4.1)	3(6.4)	
Headache D	0	46(93.9)	44(93.6)	0.958
	1	3(6.1)	3(6.4)	
Headache E	0	47(95.9)	43(91.5)	0.370
	1	2(4.1)	4(8.5)	
Skin toxicity A	0	49(100)	47(100)	NA
Skin toxicity B	0	38(77.6)	40(85.1)	0.343
	1	11(22.4)	7(14.9)	
Skin toxicity C	0	45(91.8)	44(93.6)	0.737
	1	4(8.2)	3(6.4)	
Skin toxicity D	0	43(87.8)	42(89.4)	0.805
	1	6(12.2)	5(10.6)	
Skin toxicity E	0	43(87.8)	41(91.1)	0.598
	1	6(12.2)	4(8.9)	
Peripheral neuropathy A	0	49(100)	47(100)	NA
Peripheral neuropathy B	0	46(93.9)	45(95.7)	0.681
	1	3(6.1)	2(4.3)	
Peripheral neuropathy C	0	44(89.8)	45(95.7)	0.262
	1	5(10.2)	2(4.3)	
Peripheral neuropathy D	0	46(93.9)	46(97.9)	0.328
	1	3(6.1)	1(2.1)	
Peripheral neuropathy E	0	44(89.8)	45(97.8)	0.108
	1	5(10.2)	1(2.2)	

Data expressed as n (%). NA = non-applicable. Toxicity was assessed at five time points (A, B, C, D, E) among the genotype group data analyses, where A represents the period before administering the chemotherapy, B means after taking the first cycle of chemotherapy, C means after taking the second cycle of chemotherapy, D means after taking the third cycle of chemotherapy, and E means after taking the fourth cycle of chemotherapy. The occurrence of adverse effects was categorized as follows: A score of 0 indicated that the side effect did not appear at any of the five recorded time points before or after treatment. A score of 1 was assigned if the side effect was absent before treatment but emerged during the course of treatment. A score of 2 indicated that the side effect was already present before treatment, regardless of whether it changed after treatment. A score of 3 was used if the side effect was persistent both before and after treatment. Lastly, a score of 4 signified that the side effect was absent before treatment but appeared consistently after each chemotherapy cycle.

**Table 14:** Hematological toxicity among the same genotype group data analysis using two-way with one-repeated-measure ANOVA test results.

CYP2C19	Anemia Grade	Thrombocytopenia Grade	Leukopenia Grade	Neutropenia Grade	Lymphocytopenia Grade
**CC A**	0.19 ± 0.07 A	0 ± 0 A	0 ± 0 B	0 ± 0 C	0 ± 0 C
**CC B**	0.31 ± 0.08 A	0.03 ± 0.02 A	0.09 ± 0.04 AB	0.16 ± 0.07 BC	0.09 ± 0.04 BC
**CC C**	0.41 ± 0.08 A	0.03 ± 0.02 A	0.16 ± 0.05 AB	0.72 ± 0.15 A	0.22 ± 0.07 ABC
**CC D**	0.45 ± 0.09 A	0 ± 0 A	0.19 ± 0.06 AB	0.16 ± 0.06 BC	0.27 ± 0.07 ABC
**CC E**	0.5 ± 0.1 A	0.05 ± 0.03 A	0.23 ± 0.09 A	0.61 ± 0.17 AB	0.5 ± 0.11 A
**CT+TT A**	0.22 ± 0.09 A	0 ± 0 A	0 ± 0 AB	0 ± 0 C	0 ± 0 BC
**CT+TT B**	0.28 ± 0.1 A	0.03 ± 0.03 A	0.03 ± 0.03 AB	0.06 ± 0.06 BC	0.16 ± 0.09 ABC
**CT+TT C**	0.47 ± 0.13 A	0.03 ± 0.03 A	0.03 ± 0.03 AB	0.47 ± 0.2 ABC	0.16 ± 0.08 ABC
**CT+TT D**	0.47 ± 0.12 A	0.03 ± 0.03 A	0.19 ± 0.09 AB	0.06 ± 0.06 BC	0.41 ± 0.12 AB
**CT+TT E**	0.56 ± 0.14 A	0.06 ± 0.06 A	0.06 ± 0.06 AB	0.34 ± 0.18 ABC	0.41 ± 0.12 AB
***ALDH3A1***	**Anemia grade**	**Thrombocytopenia grade**	**Leukopenia grade**	**Neutropenia grade**	**Lymphocytopenia grade**
**GC+CC A**	0.26 ± 0.09 AB	0 ± 0 D	0 ± 0 A	0 ± 0 C	0 ± 0 A
**GC+CC B**	0.26 ± 0.08 AB	0.04 ± 0.03 AB	0.06 ± 0.03 A	0.12 ± 0.07 BC	0.08 ± 0.05 A
**GC+CC C**	0.36 ± 0.09 AB	0.02 ± 0.02 AB	0.1 ± 0.04 A	0.7 ± 0.17 C	0.14 ± 0.06 A
**GC+CC D**	0.38 ± 0.1 AB	0 ± 0 AB	0.1 ± 0.05 A	0.06 ± 0.04 BC	0.3 ± 0.08 A
**GC+CC E**	0.44 ± 0.11 AB	0.02 ± 0.02 BCD	0.08 ± 0.05 A	0.38 ± 0.15 C	0.38 ± 0.1 A
**GG A**	0.13 ± 0.07 B	0 ± 0 CD	0 ± 0 A	0 ± 0 C	0 ± 0 A
**GG B**	0.35 ± 0.09 AB	0.02 ± 0.02 AB	0.09 ± 0.05 A	0.13 ± 0.08 A	0.15 ± 0.06 A
**GG C**	0.5 ± 0.11 AB	0.04 ± 0.03 A	0.13 ± 0.06 A	0.57 ± 0.16 C	0.26 ± 0.1 A
**GG D**	0.54 ± 0.11 AB	0.02 ± 0.02 AB	0.28 ± 0.09 A	0.2 ± 0.09 AB	0.33 ± 0.09 A
**GG E**	0.61 ± 0.12 A	0.09 ± 0.06 ABC	0.28 ± 0.12 A	0.67 ± 0.21 C	0.57 ± 0.13 A
***SLC22A16***	**Anemia grade**	**Thrombocytopenia grade**	**Leukopenia grade**	**Neutropenia grade**	**Lymphocytopenia grade**
**AA A**	0.15 ± 0.07 A	0 ± 0 A	0 ± 0 B	0 ± 0 D	0 ± 0 C
**AA B**	0.3 ± 0.09 A	0.04 ± 0.03 A	0.06 ± 0.04 AB	0.13 ± 0.07 BCD	0.15 ± 0.06 BC
**AA C**	0.43 ± 0.09 A	0.02 ± 0.02 A	0.13 ± 0.05 AB	0.68 ± 0.18 A	0.21 ± 0.07 ABC
**AA D**	0.47 ± 0.11 A	0.02 ± 0.02 A	0.19 ± 0.07 AB	0.13 ± 0.07 BCD	0.28 ± 0.08 ABC
**AA E**	0.57 ± 0.11 A	0.06 ± 0.05 A	0.06 ± 0.05 AB	0.66 ± 0.2 AB	0.53 ± 0.12 A
**GG+GA A**	0.24 ± 0.09 A	0 ± 0 A	0 ± 0 B	0 ± 0 D	0 ± 0 C
**GG+GA B**	0.31 ± 0.09 A	0.02 ± 0.02 A	0.08 ± 0.05 AB	0.12 ± 0.08 CD	0.08 ± 0.05 BC
**GG+GA C**	0.43 ± 0.1 A	0.04 ± 0.03 A	0.1 ± 0.05 AB	0.59 ± 0.16 ABC	0.18 ± 0.08 BC
**GG+GA D**	0.45 ± 0.1 A	0 ± 0 A	0.18 ± 0.08 AB	0.12 ± 0.07 CD	0.35 ± 0.09 AB
**GG+GA E**	0.47 ± 0.12 A	0.04 ± 0.04 A	0.29 ± 0.11 A	0.39 ± 0.16 ABCD	0.41 ± 0.11 AB
***ABCB1***	**Anemia grade**	**Thrombocytopenia grade**	**Leukopenia grade**	**Neutropenia grade**	**Lymphocytopenia grade**
**GA+AA A**	0.12 ± 0.06 B	0 ± 0 A	0 ± 0 A	0 ± 0 D	0 ± 0 C
**GA+AA B**	0.29 ± 0.08 AB	0.02 ± 0.02 A	0.04 ± 0.03 A	0.12 ± 0.08 BCD	0.08 ± 0.04 BC
**GA+AA C**	0.37 ± 0.09 AB	0.02 ± 0.02 A	0.04 ± 0.03 A	0.59 ± 0.17 ABC	0.12 ± 0.05 BC
**GA+AA D**	0.31 ± 0.08 AB	0 ± 0 A	0.12 ± 0.06 A	0.08 ± 0.05 CD	0.22 ± 0.08 BC
**GA+AA E**	0.39 ± 0.09 AB	0 ± 0 A	0.14 ± 0.07 A	0.43 ± 0.16 ABCD	0.35 ± 0.09 AB
**GG A**	0.28 ± 0.1 AB	0 ± 0 A	0 ± 0 A	0 ± 0 D	0 ± 0 C
**GG B**	0.32 ± 0.1 AB	0.04 ± 0.03 A	0.11 ± 0.05 A	0.13 ± 0.07 BCD	0.15 ± 0.07 BC
**GG C**	0.49 ± 0.11 AB	0.04 ± 0.03 A	0.19 ± 0.07 A	0.68 ± 0.18 A	0.28 ± 0.1 ABC
**GG D**	0.62 ± 0.12 A	0.02 ± 0.02 A	0.26 ± 0.09 A	0.17 ± 0.08 ABCD	0.4 ± 0.09 AB
**GG E**	0.66 ± 0.13 A	0.11 ± 0.06 A	0.21 ± 0.11 A	0.62 ± 0.2 AB	0.6 ± 0.14 A

Data expressed as mean ± standard deviation (SD). Shared letters represent non-significant results while different letters represent significant results (*p*-value < 0.05). Toxicity was assessed at five time points (A, B, C, D, E) among the genotype group data analyses, where A represents the period before administering the chemotherapy, B means after taking the first cycle of chemotherapy, C means after taking the second cycle of chemotherapy, D means after taking the third cycle of chemotherapy, and E means after taking the fourth cycle of chemotherapy.

**Table 15:** GIT toxicity among the same genotype group data analysis using two-way with one-repeated-measure ANOVA test results.

CYP2C19	Nausea Grade	Vomiting Grade	Diarrhea Grade	Constipation Grade	Mucositis Grade	Stomachache Grade
**CC A**	0 ± 0 E	0 ± 0 C	0 ± 0 A	0 ± 0 B	0 ± 0 B	0 ± 0 A
**CC B**	0.27 ± 0.06 BC	0.61 ± 0.11 A	0.14 ± 0.06 A	0.22 ± 0.06 AB	0.23 ± 0.05 A	0.16 ± 0.05 A
**CC C**	1 ± 0 A	0.59 ± 0.11 A	0.16 ± 0.06 A	0.28 ± 0.07 A	0.2 ± 0.05 A	0.11 ± 0.04 A
**CC D**	0.16 ± 0.05 CDE	0.41 ± 0.08 AB	0.13 ± 0.06 A	0.19 ± 0.05 AB	0.22 ± 0.05 A	0.09 ± 0.04 A
**CC E**	0.14 ± 0.04 CDE	0.5 ± 0.1 A	0.11 ± 0.05 A	0.22 ± 0.07 AB	0.2 ± 0.05 A	0.14 ± 0.04 A
**CT+TT A**	0 ± 0 DE	0 ± 0 BC	0 ± 0 A	0 ± 0 AB	0 ± 0 AB	0 ± 0 A
**CT+TT B**	0.41 ± 0.09 B	0.41 ± 0.13 ABC	0.19 ± 0.09 A	0.28 ± 0.09 AB	0.13 ± 0.06 AB	0.19 ± 0.07 A
**CT+TT C**	1 ± 0 A	0.44 ± 0.16 ABC	0.13 ± 0.07 A	0.19 ± 0.08 AB	0.16 ± 0.07 AB	0.13 ± 0.06 A
**CT+TT D**	0.22 ± 0.07 BCD	0.5 ± 0.15 AB	0.06 ± 0.04 A	0.19 ± 0.11 AB	0.13 ± 0.06 AB	0.16 ± 0.07 A
**CT+TT E**	0.19 ± 0.07 BCDE	0.28 ± 0.09 ABC	0.06 ± 0.04 A	0.13 ± 0.07 AB	0.06 ± 0.04 AB	0.13 ± 0.06 A
***ALDH3A1***	**Nausea grade**	**Vomiting grade**	**Diarrhea grade**	**Constipation grade**	**Mucositis grade**	**Stomachache grade**
**GC+CC A**	0 ± 0 D	0 ± 0 C	0 ± 0 A	0 ± 0 C	0 ± 0 A	0 ± 0 C
**GC+CC B**	0.34 ± 0.07 B	0.52 ± 0.11 A	0.22 ± 0.09 A	0.14 ± 0.05 ABC	0.18 ± 0.06 A	0.1 ± 0.04 ABC
**GC+CC C**	1 ± 0 A	0.48 ± 0.12 A	0.18 ± 0.08 A	0.14 ± 0.05 ABC	0.18 ± 0.05 A	0.08 ± 0.04 ABC
**GC+CC D**	0.26 ± 0.06 BC	0.42 ± 0.1 ABC	0.14 ± 0.07 A	0.08 ± 0.04 BC	0.16 ± 0.05 A	0.18 ± 0.05 ABC
**GC+CC E**	0.22 ± 0.06 BC	0.38 ± 0.09 ABC	0.12 ± 0.05 A	0.08 ± 0.04 BC	0.12 ± 0.05 A	0.08 ± 0.04 ABC
**GG A**	0 ± 0 D	0 ± 0 BC	0 ± 0 A	0 ± 0 C	0 ± 0 A	0 ± 0 BC
**GG B**	0.28 ± 0.07 BC	0.57 ± 0.13 A	0.09 ± 0.04 A	0.35 ± 0.08 AB	0.22 ± 0.06 A	0.24 ± 0.06 A
**GG C**	1 ± 0 A	0.61 ± 0.13 A	0.11 ± 0.06 A	0.37 ± 0.1 A	0.2 ± 0.06 A	0.15 ± 0.05 ABC
**GG D**	0.09 ± 0.04 CD	0.46 ± 0.1 AB	0.07 ± 0.04 A	0.3 ± 0.09 AB	0.22 ± 0.06 A	0.04 ± 0.03 ABC
**GG E**	0.09 ± 0.04 CD	0.48 ± 0.11 A	0.07 ± 0.04 A	0.3 ± 0.1 AB	0.2 ± 0.06 A	0.2 ± 0.06 AB
***SLC22A16***	**Nausea grade**	**Vomiting grade**	**Diarrhea grade**	**Constipation grade**	**Mucositis grade**	**Stomachache grade**
**AA A**	0 ± 0 C	0 ± 0 B	0 ± 0 A	0 ± 0 B	0 ± 0 B	0 ± 0 B
**AA B**	0.32 ± 0.07 B	0.62 ± 0.12 A	0.09 ± 0.04 A	0.17 ± 0.06 AB	0.23 ± 0.06 A	0.21 ± 0.06 A
**AA C**	1 ± 0 A	0.47 ± 0.12 A	0.09 ± 0.05 A	0.19 ± 0.06 AB	0.21 ± 0.06 AB	0.13 ± 0.05 AB
**AA D**	0.19 ± 0.06 BC	0.43 ± 0.1 AB	0.06 ± 0.04 A	0.15 ± 0.05 AB	0.23 ± 0.06 A	0.15 ± 0.05 AB
**AA E**	0.15 ± 0.05 BC	0.38 ± 0.1 AB	0.06 ± 0.04 A	0.09 ± 0.04 AB	0.21 ± 0.06 AB	0.17 ± 0.06 AB
**GG+GA A**	0 ± 0 C	0 ± 0 B	0 ± 0 A	0 ± 0 B	0 ± 0 B	0 ± 0 B
**GG+GA B**	0.31 ± 0.07 B	0.47 ± 0.11 A	0.22 ± 0.09 A	0.31 ± 0.08 A	0.17 ± 0.05 AB	0.12 ± 0.05 AB
**GG+GA C**	1 ± 0 A	0.61 ± 0.13 A	0.2 ± 0.08 A	0.31 ± 0.09 A	0.16 ± 0.05 AB	0.1 ± 0.04 AB
**GG+GA D**	0.16 ± 0.05 BC	0.45 ± 0.1 A	0.14 ± 0.07 A	0.22 ± 0.08 AB	0.14 ± 0.05 AB	0.08 ± 0.04 AB
**GG+GA E**	0.16 ± 0.05 BC	0.47 ± 0.1 A	0.12 ± 0.06 A	0.29 ± 0.09 AB	0.1 ± 0.04 AB	0.1 ± 0.04 AB
***ABCB1***	**Nausea grade**	**Vomiting grade**	**Diarrhea grade**	**Constipation grade**	**Mucositis grade**	**Stomachache grade**
**GA+AA A**	0 ± 0 D	0 ± 0 C	0 ± 0 A	0 ± 0 A	0 ± 0C	0 ± 0 A
**GA+AA B**	0.24 ± 0.06 BC	0.47 ± 0.12 A	0.16 ± 0.07 A	0.27 ± 0.07 A	0.23 ± 0.06 A	0.16 ± 0.05 A
**GA+AA C**	1 ± 0 A	0.63 ± 0.14 A	0.18 ± 0.07 A	0.29 ± 0.09 A	0.22 ± 0.06 AB	0.1 ± 0.04 A
**GA+AA D**	0.14 ± 0.05 CD	0.51 ± 0.11 A	0.1 ± 0.04 A	0.18 ± 0.06 A	0.2 ± 0.06 ABC	0.1 ± 0.04 A
**GA+AA E**	0.12 ± 0.05 CD	0.41 ± 0.11 ABC	0.1 ± 0.04 A	0.2 ± 0.08 A	0.16 ± 0.05 ABC	0.12 ± 0.05 A
**GG A**	0 ± 0 D	0 ± 0 BC	0 ± 0 A	0 ± 0 A	0 ± 0 BC	0 ± 0 A
**GG B**	0.38 ± 0.07 B	0.62 ± 0.12 A	0.15 ± 0.07 A	0.21 ± 0.07 A	0.17 ± 0.06 ABC	0.17 ± 0.06 A
**GG C**	1 ± 0 A	0.45 ± 0.11 AB	0.11 ± 0.07 A	0.21 ± 0.07 A	0.15 ± 0.05 ABC	0.13 ± 0.05 A
**GG D**	0.21 ± 0.06 BC	0.36 ± 0.08 ABC	0.11 ± 0.07 A	0.19 ± 0.08 A	0.17 ± 0.06 ABC	0.13 ± 0.05 A
**GG E**	0.19 ± 0.06 BCD	0.45 ± 0.1 AB	0.09 ± 0.05 A	0.17 ± 0.06 A	0.15 ± 0.05 ABC	0.15 ± 0.05 A

Data expressed as mean ± standard deviation (SD). Shared letters represent non-significant results while different letters represent significant results (*p*-value < 0.05). Toxicity was assessed at five time points (A, B, C, D, E) among the genotype group data analyses, where A represents the period before administering the chemotherapy, B means after taking the first cycle of chemotherapy, C means after taking the second cycle of chemotherapy, D means after taking the third cycle of chemotherapy, and E means after taking the fourth cycle of chemotherapy.

**Table 16:** Miscellaneous toxicities among the same genotypes group data analysis using two-way with one-repeated-measure ANOVA test results.

CYP2C19	Fever Grade	Fatigue Grade	Amenorrhea Grade	Alopecia Grade	Headache Grade	Skin Toxicity Grade	Peripheral Neuropathy Grade
**CC A**	0 ± 0 A	0 ± 0 C	0 ± 0 B	0 ± 0 J	0 ± 0 A	0 ± 0 B	0 ± 0 A
**CC B**	0.08 ± 0.03 A	0.31 ± 0.06 A	0.11 ± 0.04 A	1 ± 0 H	0.11 ± 0.04 A	0.2 ± 0.05 A	0.06 ± 0.03 A
**CC C**	0.06 ± 0.03 A	0.22 ± 0.05 AB	0.03 ± 0.02 AB	1 ± 0 G	0.05 ± 0.03 A	0.08 ± 0.03 AB	0.09 ± 0.04 A
**CC D**	0.05 ± 0.03 A	0.17 ± 0.05 ABC	0.02 ± 0.02 AB	1 ± 0 F	0.05 ± 0.03 A	0.14 ± 0.04 AB	0.06 ± 0.03 A
**CC E**	0.03 ± 0.02 A	0.16 ± 0.05 ABC	0.03 ± 0.02 AB	1 ± 0 E	0.06 ± 0.03 A	0.11 ± 0.04 AB	0.08 ± 0.03 A
**CT+TT A**	0 ± 0 A	0 ± 0 BC	0 ± 0 AB	0 ± 0 I	0 ± 0 A	0 ± 0 B	0 ± 0 A
**CT+TT B**	0.03 ± 0.03 A	0.41 ± 0.09 A	0.06 ± 0.04 AB	1 ± 0 C	0.13 ± 0.06 A	0.16 ± 0.07 AB	0.03 ± 0.03 A
**CT+TT C**	0.03 ± 0.03 A	0.28 ± 0.08 AB	0.06 ± 0.04 AB	1 ± 0 B	0.06 ± 0.04 A	0.06 ± 0.04 AB	0.03 ± 0.03 A
**CT+TT D**	0.06 ± 0.04 A	0.25 ± 0.08 ABC	0.06 ± 0.04 AB	1 ± 0 A	0.09 ± 0.05 A	0.06 ± 0.04 AB	0 ± 0 A
**CT+TT E**	0 ± 0 A	0.22 ± 0.07 ABC	0.03 ± 0.03 AB	1 ± 0 D	0.06 ± 0.04 A	0.09 ± 0.05 AB	0.03 ± 0.03 A
***ALDH3A1***	**Fever grade**	**Fatigue grade**	**Amenorrhea grade**	**Alopecia grade**	**Headache grade**	**skin toxicity grade**	**Peripheral neuropathy grade**
**GC+CC A**	0 ± 0 B	0 ± 0 C	0 ± 0 B	0 ± 0 J	0 ± 0 A	0 ± 0 B	0 ± 0 B
**GC+CC B**	0 ± 0 B	0.22 ± 0.06 BC	0.06 ± 0.03 AB	1 ± 0 H	0.12 ± 0.05 A	0.22 ± 0.06 A	0.08 ± 0.04 AB
**GC+CC C**	0 ± 0 B	0.18 ± 0.05 BC	0.04 ± 0.03 AB	1 ± 0 G	0.04 ± 0.03 A	0.1 ± 0.04 AB	0.14 ± 0.05 A
**GC+CC D**	0 ± 0 B	0.16 ± 0.05 BC	0.04 ± 0.03 AB	1 ± 0 E	0.04 ± 0.03 A	0.08 ± 0.04 AB	0.04 ± 0.03 AB
**GC+CC E**	0 ± 0 B	0.16 ± 0.05 BC	0.04 ± 0.03 AB	1 ± 0 D	0.04 ± 0.03 A	0.16 ± 0.05 AB	0.06 ± 0.03 AB
**GG A**	0 ± 0 B	0 ± 0 C	0 ± 0 B	0 ± 0 I	0 ± 0 A	0 ± 0 B	0 ± 0 B
**GG B**	0.13 ± 0.05 A	0.48 ± 0.07 A	0.13 ± 0.05 A	1 ± 0 C	0.11 ± 0.05 A	0.15 ± 0.05 AB	0.02 ± 0.02 AB
**GG C**	0.11 ± 0.05 AB	0.3 ± 0.07 AB	0.04 ± 0.03 AB	1 ± 0 B	0.07 ± 0.04 A	0.04 ± 0.03 AB	0 ± 0 B
**GG D**	0.11 ± 0.05 AB	0.24 ± 0.06 ABC	0.02 ± 0.02 AB	1 ± 0 A	0.09 ± 0.04 A	0.15 ± 0.05 AB	0.04 ± 0.03 AB
**GG E**	0.04 ± 0.03 AB	0.2 ± 0.06 BC	0.02 ± 0.02 AB	1 ± 0 F	0.09 ± 0.04 A	0.04 ± 0.03 AB	0.07 ± 0.04 AB
***SLC22A16***	**Fever**	**Fatigue**	**Amenorrhea**	**Alopecia**	**Headache**	**Skin toxicity**	**Peripheral neuropathy**
**AA A**	0 ± 0 A	0 ± 0 B	0 ± 0 A	0 ± 0 I	0 ± 0 A	0 ± 0 B	0 ± 0 A
**AA B**	0.09 ± 0.04 A	0.34 ± 0.07 A	0.09 ± 0.04 A	1 ± 0 H	0.15 ± 0.05 A	0.17 ± 0.06 AB	0.06 ± 0.04 A
**AA C**	0.04 ± 0.03 A	0.28 ± 0.07 A	0.02 ± 0.02 A	1 ± 0 G	0.06 ± 0.04 A	0.09 ± 0.04 AB	0.06 ± 0.04 A
**AA D**	0.04 ± 0.03 A	0.21 ± 0.06 AB	0.02 ± 0.02 A	1 ± 0 F	0.06 ± 0.04 A	0.11 ± 0.05 AB	0.06 ± 0.04 A
**AA E**	0.02 ± 0.02 A	0.17 ± 0.06 AB	0.02 ± 0.02 A	1 ± 0 E	0.09 ± 0.04 A	0.09 ± 0.04 AB	0.04 ± 0.03 A
**GG+GA A**	0 ± 0 A	0 ± 0B	0 ± 0 A	0 ± 0 J	0 ± 0 A	0 ± 0 B	0 ± 0 A
**GG+GA B**	0.04 ± 0.03 A	0.35 ± 0.07 A	0.1 ± 0.04 A	1 ± 0 D	0.08 ± 0.04 A	0.2 ± 0.06 A	0.04 ± 0.03 A
**GG+GA C**	0.06 ± 0.03 A	0.2 ± 0.06 AB	0.06 ± 0.03 A	1 ± 0 C	0.04 ± 0.03 A	0.06 ± 0.03 AB	0.08 ± 0.04 A
**GG+GA D**	0.06 ± 0.03 A	0.18 ± 0.06 AB	0.04 ± 0.03 A	1 ± 0 B	0.06 ± 0.03 A	0.12 ± 0.05 AB	0.02 ± 0.02 A
**GG+GA E**	0.02 ± 0.02 A	0.18 ± 0.06 AB	0.04 ± 0.03 A	1 ± 0 A	0.04 ± 0.03 A	0.12 ± 0.05 AB	0.08 ± 0.04 A
***ABCB1***	**Fever**	**Fatigue**	**Amenorrhea**	**Alopecia**	**Headache**	**Skin toxicity**	**Peripheral neuropathy**
**GA+AA A**	0 ± 0 A	0 ± 0 B	0 ± 0 A	0 ± 0 J	0 ± 0 A	0 ± 0 B	0 ± 0 A
**GA+AA B**	0.08 ± 0.04 A	0.37 ± 0.07 A	0.12 ± 0.05 A	1 ± 0 H	0.12 ± 0.05 A	0.22 ± 0.06 A	0.06 ± 0.03 A
**GA+AA C**	0.04 ± 0.03 A	0.22 ± 0.06 AB	0.04 ± 0.03 A	1 ± 0 G	0.04 ± 0.03 A	0.08 ± 0.04 AB	0.1 ± 0.04 A
**GA+AA D**	0.06 ± 0.03 A	0.22 ± 0.06 AB	0.04 ± 0.03 A	1 ± 0 F	0.06 ± 0.03 A	0.12 ± 0.05 AB	0.06 ± 0.03 A
**GA+AA E**	0.04 ± 0.03 A	0.22 ± 0.06 AB	0.04 ± 0.03 A	1 ± 0 E	0.04 ± 0.03 A	0.12 ± 0.05 AB	0.1 ± 0.04 A
**GG A**	0 ± 0 A	0 ± 0 B	0 ± 0 A	0 ± 0 I	0 ± 0	0 ± 0 B	0 ± 0 A
**GG B**	0.04 ± 0.03 A	0.32 ± 0.07 A	0.06 ± 0.04 A	1 ± 0 D	0.11 ± 0.05	0.15 ± 0.05 AB	0.04 ± 0.03 A
**GG C**	0.06 ± 0.04 A	0.26 ± 0.06 A	0.04 ± 0.03 A	1 ± 0 C	0.06 ± 0.04	0.06 ± 0.04 AB	0.04 ± 0.03 A
**GG D**	0.04 ± 0.03 A	0.17 ± 0.06 AB	0.02 ± 0.02 A	1 ± 0 B	0.06 ± 0.04	0.11 ± 0.05 AB	0.02 ± 0.02 A
**GG E**	0 ± 0 A	0.13 ± 0.05 AB	0.02 ± 0.02 A	1 ± 0 A	0.09 ± 0.04	0.09 ± 0.04 AB	0.02 ± 0.02 A

Data expressed as mean ± standard deviation (SD). Shared letters represent non-significant results while different letters represent significant results (*p*-value < 0.05). Toxicity was assessed at five time points (A, B, C, D, E) among the genotype group data analyses, where A represents the period before administering the chemotherapy, B means after taking the first cycle of chemotherapy, C means after taking the second cycle of chemotherapy, D means after taking the third cycle of chemotherapy, and E means after taking the fourth cycle of chemotherapy.

## Data Availability

Data are available from the corresponding author (H.A.A.M.) upon reasonable request due to patients privacy.

## Appendix A

**Table A1:** Count and percentage of each genotype group among the 96 patients along the *CYP2C19*, *ALDH3A1*, *SLC22A16*, and *ABCB1* genes.

*CYP2C19*	Count (%)	*CYP2C19*	Count (%)
CC	64 (66.67)	CC	64 (66.67)
CT	28 (29.17)	CT+TT	32 (33.33)
TT	4 (4.17)	N=	96 (100)
N=	96 (100)		
*ALDH3A1*	Count (%)	*ALDH3A1*	Count (%)
CC	11 (11.46)	GC+CC	50 (52.08)
GC	39 (40.63)	GG	46 (47.92)
GG	46 (47.92)	N=	96 (100)
N=	96 (100)		
*SLC22A16*	Count (%)	*SLC22A16*	Count (%)
AA	47 (48.96)	AA	47 (48.96)
GA	38 (39.58)	GG+GA	49 (51.04)
GG	11 (11.46)	N=	96 (100)
N=	96 (100)		
*ABCB1*	Count (%)	*ABCB1*	Count (%)
AA	11 (11.46)	GA+AA	47(48.96)
GA	38 (39.58)	GG	49(51.04)
GG	47 (48.96)	N=	96 (100)
N=	96 (100)		

Data expressed as n (%).
